# Evaluation of Iron-Lignin Particles Obtained by the Nanoprecipitation Method: Influence of Process Conditions on Morphology, Structure, and Properties

**DOI:** 10.1021/acsomega.6c00916

**Published:** 2026-06-04

**Authors:** Jardel Machado de Lima, Beatriz Alves Biscola, Sônia F. Zawadzki, Luiz Pereira Ramos, Daniel Eiras

**Affiliations:** † 28122 Federal University of Parana, Graduate Program in Chemical Engineering, Curitiba 80.060-000, Brazil; ‡ 503031 Federal University of Parana, Graduate Program in Materials Engineering and Science, Curitiba 80.060-000, Brazil; § Federal University of Parana, Graduate Program in Chemistry, Curitiba 80.060-000, Brazil

## Abstract

Lignin nanoparticles (LNPs) are important biobased materials that have been considered for several applications such as antioxidants, UV protectants, heavy metal absorption, antimicrobials, drug carriers, gene delivery systems, encapsulation of molecules, biocatalysts, supercapacitors, tissue engineering, hybrid nanocomposites, and wound dressing. Moreover, lignin-functionalized metal nanoparticles can be applied for different catalytic reactions. Despite their technological importance, the synthesis of lignin and lignin-functionalized metal nanoparticles remains an unexplored subject, in particular the methods to produce uniform spherical particles. In this context, the aim of this work is to analyze the effect of different variables on the morphology, particle size, particle size distribution, and properties of two different kraft lignins. Based on the concept of chain coiling and decoiling, two different solvents, four different temperatures, and two kinds of kraft lignins were tested. A nanoprecipitation method was applied, resulting in iron-lignin particles with different morphologies. The use of a THF/water mixture as a solvent resulted in spherical lignin particles, and the use of DMF/water resulted in elongated shapeless particles. Higher temperatures (40 °C) led to larger particles. Also, softwood lignin produced larger particles than hardwood lignin. FTIR-ATR analysis showed that the attenuation of signal peaks between 1700 and 1030 cm^–1^ and the appearance of a new band at 1652 cm^–1^ indicate the formation of iron complexes. Also, lignin glass transition temperatures are reduced with the incorporation of iron. Zeta potential indicates that softwood iron/lignin particles have higher colloidal stability than neat lignin nanoparticles. Three iron/lignin nanoparticles were tested to evaluate their ability to oxidize methylene blue in the presence of hydrogen peroxide. The results show that neat lignin, especially softwood lignin, is very effective at reducing methylene blue (MB) concentration in solution. Additionally, iron-lignin nanoparticles might be degrading methylene blue, which is evidenced by a peak shift in UV–vis spectra, and removing this new reaction product from solution by adsorption. Finally, a mechanism of nanoparticle formation by nanoprecipitation is proposed. The mechanism is based on both a kinetic trap that determines the final shape of lignin nanoparticles depending on the solvency power of the solvent and the thermodynamic stability of short lignin chains that determines the recovery of nanoparticles. This scheme illustrates the solvent-driven mechanism of lignin-iron nanoparticle assembly via nanoprecipitation. In THF, lignin adopts a collapsed micellar conformation stabilized by native apolar π–π interactions, effectively hiding internal hydroxyl groups within a hydrophobic core. Conversely, the high solvating power of DMF induces an extended polymer configuration, fully exposing these reactive hydroxyls. Upon dropwise addition into an aqueous Fe^3+^ solution, rapid metal coordination and high supersaturation trigger immediate precipitation by shifting the critical aggregation concentration (THF) and overall polymer solubility (DMF). This fast precipitation acts as a kinetic trap, preventing further morphological rearrangement. Consequently, the final architecture is directly templated by the initial solvent state, recovering discrete spherical nanoparticles from THF and extended cross-linked networks from DMF. Furthermore, this rapid phase separation selectively precipitates high-molecular-weight lignin chains, retaining highly soluble, short-chain oligomers in the supernatant and explaining the macroscopic particle recovery.

## Graphical abstract

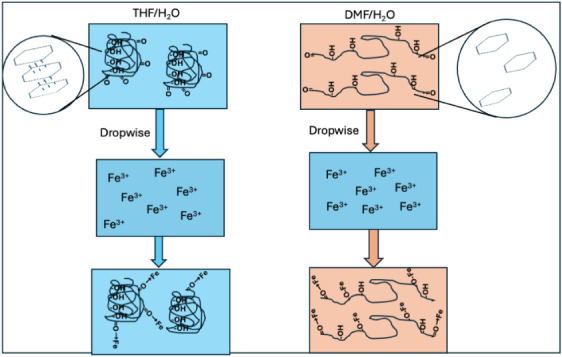

## Introduction

Metal-phenolic nanoparticles have great potential for drug delivery, bioimaging, tissue engineering, and cancer therapy.^1,2^ Polyphenols provide interaction points via hydrogen bonding, π–π stacking, hydrophobic interactions, and metal coordination.^1,3^ Moreover, iron-polyphenol nanoparticles can be applied as a external ROS (reactive oxygen species) generator via Fenton reaction for cancer therapy^4^

Lignin, a natural component extracted from black liquor, is an important source of polyphenols that has antioxidant and antimicrobial activity that can be applied for medical applications.^5^ Also, lignin possesses an interesting adsorption capacity, making it effective for removing heavy metal ions by complexation^6^ and polyphenol-metal nanoparticle synthesis.^7^ This substance is the second most abundant biopolymer in nature, accounting for 15% of all planet biomass.^8^

Lignin can be obtained from coniferous trees (softwood lignin) and dicotyledonous trees (hardwood lignins).^9^ Softwood woods typically have between 23 and 33 wt % of lignin, and hardwood woods have 16–25 wt % of lignin.^10^ Softwoods contain more G (guaiacyl) groups^11^—94 wt % coniferyl alcohol, 1 wt % sinapyl alcohol, and 5 wt % p-coumaryl alcohol.^12^ Hardwoods are mainly composed of guaiacyl (G) and syringyl (S) precursors. The syringyl concentration varies between 20 and 60 wt %.^10^ Additionally, the cellulose extraction process results in different lignins with different compositions and molecular weights. Kraft pulping, which produces Kraft lignin, is the most common and well-known cellulose extraction process in industries worldwide.^13^

Studies to obtain lignin nanoparticles (LNPs) from different sources,^14,15^ and their applications in mixtures, copolymers, and composites that seek process costs and pollution reduction are easily found in the literature.^16^ Metal–organic coordinative compounds, produced by coordination reactions between lignin and metal salts,^17^ show that there are two main approaches to obtaining LNPs: top-down techniques, which use physical methods like grinding to break down a macroscopic substance into smaller units; or bottom-up techniques, where small particles self-assemble to form larger structures.^18^ The process of self-association consists in the formation of organized structures from disorganized subunits, without the interference of external forces and is governed by internal forces and interactions within the system itself, to obtain a state of equilibrium or minimization of free energy,^19,20^

In the preparation of lignin nanoparticles (LNPs) using bottom-up approaches, two solvents are typically employed: a solvent with high affinity for lignin (good solvent) and a second solvent with low affinity for lignin (poor solvent or antisolvent).^21,22^ When a nonsolvent (water) is added to a lignin solution (or vice versa), the change in solvent quality induces a supersaturated state, which drives particle formation through nucleation and growth.^22,23^ Once the solute concentration exceeds the equilibrium saturation concentration of the solvent–antisolvent system, the solution becomes supersaturated, and nucleation occurs when a critical supersaturation level is reached.^22^

Upon introduction of the organic lignin solution into the aqueous phase, an interface is formed between the two liquids. A rapid diffusion of water (the nonsolvent) occurs across this interface into the organic phase, inducing a high local supersaturation and triggering the self-association of lignin molecules and the formation of initial aggregates. Continued diffusion of water through this initial layer sustains the self-association process and promotes particle growth.^24^ The subsequent evolution of these particles, whether by molecular growth or aggregation, is controlled by the diffusion of lignin molecules and the overall mixing dynamics, as increased collision frequency and molecular interactions favor the growth mechanism.^25^

An important self-association method that results in LNPs is nanoprecipitation.^26^ The difference in solubility of lignin in the organic solvent and the nonsolvent (usually water) causes lignin molecules to self-assemble into spheres due to intermolecular secondary interactions (hydrophobic, van der Waals, π–π stacking, and hydrogen bonding).^27–29^ The formation of LNPs is attributed to hydrophobic interactions and π–π stacking of aromatic rings; additional noncovalent interactions, such as intra- and intermolecular hydrogen bonding and van der Waals forces, contribute to the stabilization of the resulting aggregates.^30^ Depending on the solvating capacity of the solvent, lignin can precipitate to form spherical nanoparticles or shapeless elongated particles.^23,31^

To move beyond discussing the underlying mechanism and toward predictably engineering lignin/iron nanoparticles, a systematic study is needed to clarify the influence of key process variables (temperature, lignin/solvent affinity, and lignin type) on particle morphology and size.

Therefore, this work investigated the effect of different variables on the morphology, size, and size distribution of particles obtained with two different solvent mixtures, four different temperatures, and two kinds of Kraft lignin. Both softwood and hardwood lignins were dissolved in THF/water and DMF/water, and the solutions were dripped into an iron nitrate water solution. The resulting nanoparticles were characterized by SEM and/or TEM, FTIR, DLS, and zeta potential, and selected particles were tested to verify their ability to degrade methylene blue by the Fenton reaction. Finally, a mechanism for nanoparticle formation by nanoprecipitation is proposed. The mechanism is based on both a kinetic trap that determines the final shape of lignin nanoparticles depending on the solvency power of the solvent and the thermodynamic stability of short lignin chains that determines the recovery of nanoparticles.

## Experimental Section

### Materials

Kraft LignoForce lignins were kindly provided by FP Innovations (Pointe-Claire, QC, Canada). Softwood lignin was extracted from black liquor obtained from wood mills using lodgepole pine, spruce, and fir (wood mix ratio of 74:25:2), and hardwood lignin was extracted from black liquor obtained from wood mills using aspen.^32^ Metal source was iron­(III) nitrate nonahydrate (Fe­(NO_3_)_3_.9H_2_O) from Synth. Solvents are NEON *N*,*N*-dimethylformamide (DMF) 99.8%, êxodo científica tetrahydrofuran (THF) P.A. ACS, and deionized water (2.12 ± 0.03 μS/cm). Hydrogen peroxide 35% and methylene blue (MB) PA from êxodo científica were used as received for MB degradation tests.

### Lignin Stock Solution

A lignin stock solution was prepared by dispersing 10 g of lignin in 1 L of a binary mix (THF/water and DMF/water −4:1 ratio in volume) and stirring for 24 h. To remove any insoluble particles, the final solution was centrifuged (15 min −4000 rpm) and vacuum filtered.

### Preparation of Iron-Lignin Nanoparticles

Lignin-iron nanoparticles were prepared by a nanoprecipitation method adapted from Chen et al.^31^ In a typical synthesis, 100 mL of lignin stock solution was added dropwise over 30 min into 900 mL of iron nitrate solution (C = 20 mmol L^–1^) under continuous stirring. The reaction mixture was maintained under controlled temperatures (0, 25, 40, and 60 °C) and stirred for 90 min. The resulting suspension was allowed to stand undisturbed for 24 h to complete precipitation. The precipitate was collected by centrifugation (4000 rpm, 10 min), followed by vacuum filtration and washing with 200 mL of deionized water to remove residual reagents. The obtained solids were vacuum-dried at 105 °C for 24 h. The nanoparticle production recovery was determined gravimetrically based on the mass of the dried precipitate.

To maintain a constant hydroxyl-to-iron (OH:Fe) molar ratio, the amount of iron nitrate was calculated based on the work of Suota and coworkers.^32^ Using their reported hydroxyl contents of 6.0 mmol g^–1^ (softwood) and 5.4 mmol g^–1^ (hardwood), the addition of 7.3296 ± 0.0797 g of iron nitrate per gram of lignin achieved the target molar ratios of 3:1 and 2.7:1, respectively.

### Attenuated Total Reflectance Fourier Transform Infrared Spectroscopy (ATR-FTIR)

Infrared spectra of lignin particles were obtained on an FTIR VERTEX 70v with attenuated total reflection, with a DLATGS detector with a KBr window. The spectra were recorded between 400 and 4000 cm^–1^ at a resolution of 4 cm^–1^ in the diffuse mode, and 32 runs by sample.

### Differential Scanning Calorimetry (DSC)

Differential Scanning Calorimetry (DSC) measurements were carried out using a NETZSCH DSC 200F3 instrument. Samples weighing between 7 and 19 mg were heated and cooled at a constant rate of 10 °C/min under a nitrogen atmosphere (flow rate: 40 mL/min). Three consecutive heating/cooling cycles were performed: first cycle —20–0 °C, then 0–200 °C, followed by cooling from 200 to 0 °C (to erase previous thermal history); second cycle—heating from 0 to 300 °C and cooling back to 0 °C (to obtain glass transition temperatures); third cycle—heating from 0 to 450 °C (to obtain pyrolysis heat). Glass transition temperature (*T*_g_) and decomposition temperatures were determined using NETZSCH Proteus software.

### Energy-Dispersive X-ray Spectroscopy (EDS) and Scanning Electron Microscopy (SEM)

SEM images were captured using two microscopes: TESCAN VEGA 3 LMU operating at 15 kV, and Philips XL-30 FEG operating at 15 kV. Chemical analysis was performed in an Oxford instruments’ 80 - mm^2^ X-Max silicon-drift detector (SDD) with AZ Tech (Advanced) software.

Iron-lignin samples were prepared by using distinct protocols to capture their respective states. The iron-lignin nanoparticles were prepared via the nanoprecipitation method described above. For the neat lignin, taking advantage of its inherent amphiphilic nature and spontaneous self-assembly into micellar structures, the sample was simply dispersed in water. Both suspensions were subsequently drop-cast onto the SEM supports and allowed to dry at room temperature prior to imaging.

### Transmission Electron Microscopy (TEM)

TEM was performed on two different pieces of equipment, JEOL JEM F200 and FEI Tecnai G2 F20 operating at 200 kV. Lignin nanoparticles were dispersed in water and dropped onto a copper grid with a Parafilm.

Iron-lignin samples were prepared by using distinct protocols to capture their respective states. The iron-lignin nanoparticles were prepared via the nanoprecipitation method described above. For the neat lignin, taking advantage of its inherent amphiphilic nature and spontaneous self-assembly into micellar structures, the sample was simply dispersed in water. Both suspensions were subsequently drop-cast onto the TEM supports and allowed to dry at room temperature prior to imaging.

### Particle Size Distribution

Particle diameters were measured from SEM and TEM images using the software ImageJ. Normal quantile–quantile and bar plots were also generated.

### Zeta Potential and Hydrodynamic Diameter

The hydrodynamic diameter of lignin particles was determined by dynamic light scattering (DLS) using a Malvern Zetasizer Nano Series analyzer. Samples were prepared by dispersing 0.5 mg of lignin in 5 mL of deionized water, followed by bath ultrasonication (7Lab) for 30 min. The dispersions were further diluted 1:10 with deionized water to achieve a final concentration of 0.01 mg/mL. Measurements were performed at 25 °C with detection angles of 173° and 12.8°. Three independent measurements were performed for each sample, and the Z-average diameter and polydispersity index (PdI) were calculated using the equipment software. For samples showing bimodal distributions, the weighted average considering both peaks was reported.

The colloidal stability of lignin particles in water was determined using the same Malvern Zetasizer Nano Series analyzer equipped with a DTS1070 capillary cell. Samples were prepared following the same protocol described for DLS analysis: 0.5 mg of material was dispersed in 5 mL of deionized water, bath ultrasonicated (7Lab) for 30 min, and diluted 1:10 to a final concentration of 0.01 mg/mL. Zeta potential values were determined at 25 °C. Three independent measurements were performed for each sample, and the average values with standard deviations were reported.

### Methylene Blue Degradation

Methylene blue aqueous solutions were prepared by dissolving 10 mg of methylene blue in 1000 mL of water to form a 10 mg/L solution. The pH of the solution was adjusted from 5 to 7 by addition of a 0.01 M sodium hydroxide solution. For the degradation tests, 3 mg of lignin nanoparticles were dispersed in deionized water with a tip ultrasound from Omini International model SONIC Ruptor 4000, 115 V/60 Hz/4 A, outlet power of 300 W, amplitude of 20%, for 2 min. Meanwhile, hydrogen peroxide was added to the MB solution, and the two solutions were mixed. The UV–vis absorbance at 664–680 nm was evaluated every 10 min during the first hour and every hour after the first hour. Background curves were obtained from pure lignin aqueous solutions and were subtracted from the results.

## Results and Discussion

### Effect of Process Variables on Iron-Lignin Particles Recovery and Appearance

We systematically investigated the influence of three key process variables—temperature, solvent type, and lignin type—on the resulting nanoparticle size, shape, and iron content. The full experimental matrix and sample codes are detailed in Table 1. A total of 16 samples were prepared, varying the type of lignin (hardwood and softwood), the nanoprecipitation temperature (0, 25, 40, and 60 °C), and two binary solvent mixtures (DMF/water and THF/water). Table S1 summarizes the input mass of all raw materials and the output mass of the resulting lignin/iron nanoparticles for each synthesis.

**Table 1:** Sample Names and Designation Codes

Sample^a^	Solvent	Lignin Type	Temperature (°C)
T-SOFT0	THF/water	Softwood	0
T-SOFT25	THF/water	Softwood	25
T-SOFT40	THF/water	Softwood	40
T-SOFT60	THF/water	Softwood	60
T-HARD0	THF/water	Hardwood	0
T-HARD25	THF/water	Hardwood	25
T-HARD40	THF/water	Hardwood	40
T-HARD60	THF/water	Hardwood	60
D-SOFT0	DMF/water	Softwood	0
D-SOFT25	DMF/water	Softwood	25
D-SOFT40	DMF/water	Softwood	40
D-SOFT60	DMF/water	Softwood	60
D-HARD0	DMF/water	Hardwood	0
D-HARD25	DMF/water	Hardwood	25
D-HARD40	DMF/water	Hardwood	40
D-HARD60	DMF/water	Hardwood	60

a D and T stand for DMF and THF, HARD and SOFT stand for the types of lignin, and the numbers refer to the temperature of the process.

Figure 1 shows the product recovery for each condition based on the mass of dried nanoparticles in relation to the initial mass of raw materials added to the reaction.

**Figure 1:**
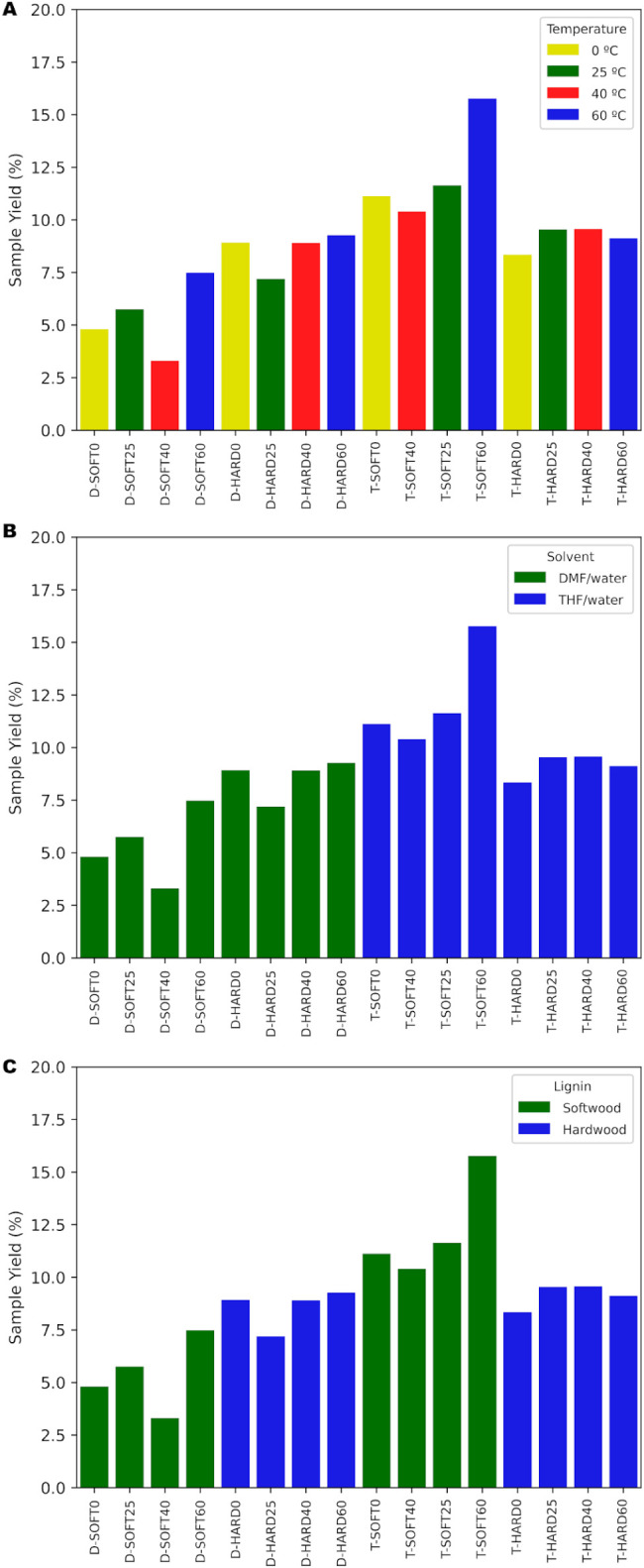
Product recovery as a of A) temperature, B) solvent, and C) lignin type .

By analyzing the product recovery for softwood lignin, the use of THF appears to result in higher product recovery as the temperature increases, compared to DMF. In contrast, for hardwood lignin, a smaller increase in product recovery is observed. Additionally, the use of DMF results in higher product recovery than that of THF.

When comparing samples obtained using DMF, the increase in temperature has a stronger effect on the product recovery of nanoparticles produced from softwood lignin. A similar trend is observed when THF is used as the solvent: increasing the temperature promotes a greater increase in product recovery for softwood lignin compared with hardwood lignin.

The product recovery was greater with the use of THF/water as a solvent and softwood lignin. For hardwood lignin, product recovery differences among samples were not conclusive, but at 25 and 40 °C, the product recovery was greater with THF/water as a solvent, while at 0 and 60 °C the product recovery was greater in the presence of DMF/water. For each lignin and solvent combination, the influence of temperature on product recovery shows that the product recovery obtained at 60 °C was greater than the product recovery obtained at 0 °C.

Therefore, when comparing the influence of solvent on each lignin type, softwood lignin exhibited significantly higher product recovery for THF/water compared to DMF/water, particularly at elevated temperatures. In contrast, for hardwood lignin, product recovery is less dependent on solvent type, with comparable product recoveries observed for DMF and THF systems. These findings suggest that the precipitation of softwood lignin is more sensitive to solvent polarity and solvation effects, whereas hardwood lignin is less affected by solvent substitution. It has been reported that THF shows preferential solvation for guaiacyl and p-hydroxyphenyl units—characteristic of softwood lignin—and hardwood lignin is less soluble in THF because of its higher content of syringyl units.^33^

The macroscopic appearance of the particles (Figure 2), particularly their color and shine, was influenced by the solvent used. The majority of particles precipitated from DMF/water solutions (samples D-HARD and D-SOFT) were black, exhibiting a discernible sheen, with only D-HARD0 being the exception. In contrast, iron/lignin nanoparticles produced from THF/water mixtures (T-SOFT and T-HARD) had different tones of brown, except for T-HARD0, which appeared black. For context, neat softwood and hardwood lignins are brown.

**Figure 2:**
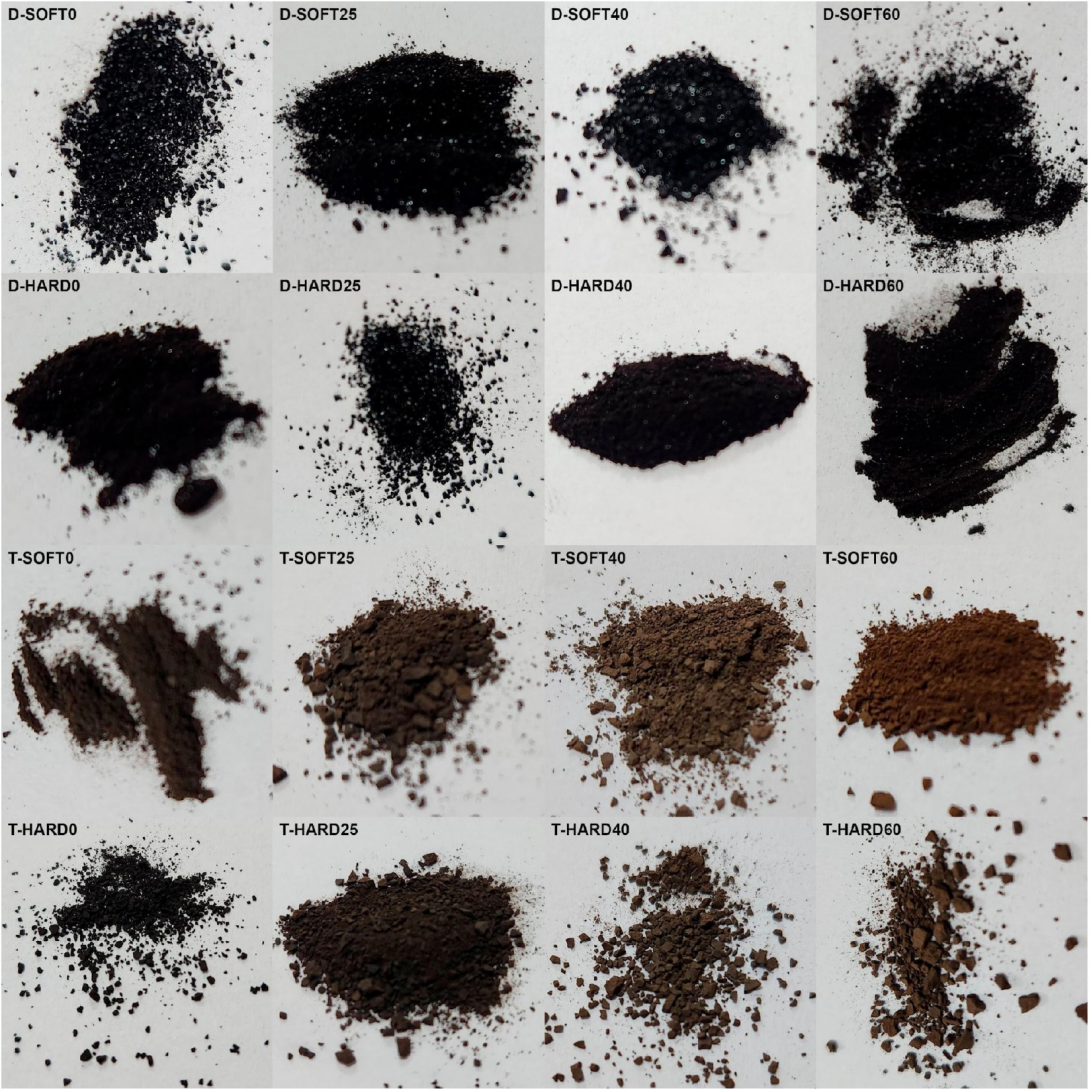
Pictures of lignin-iron nanoparticles.

### Energy-Dispersive X-ray Spectroscopy (EDS) of Particles

Energy-dispersive X-ray spectroscopy (EDS) was employed to identify the elemental composition of the iron-lignin nanoparticles (Figure 3). The analysis confirmed that all samples are primarily composed of carbon, oxygen, and iron, with sulfur also detected in all samples. As expected, hydrogen is not detected, as it is below the detection limit of the EDS technique. The presence of sulfur can be attributed to residual sulfuric acid from the Kraft process used to extract the lignin.^34^

**Figure 3:**
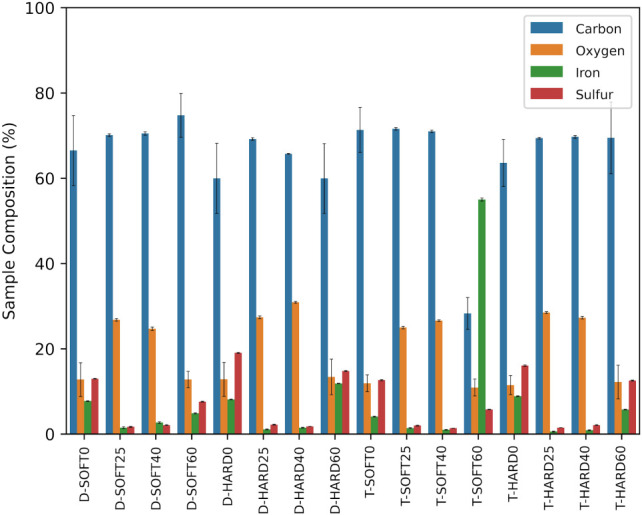
Elemental particle composition from EDS analysis.

While this technique is powerful for elemental identification, the resulting atomic percentages should be considered semiquantitative. The estimated elemental compositions for each sample are summarized in Table S2. The apparent iron content showed significant variation, ranging from approximately 1% to as high as 55%. This wide range suggests that the precipitation protocol strongly influences the final amount of iron incorporated into the nanoparticles. For context, the reported concentrations for the starting Kraft lignin are typically around 65% for carbon and 27% for oxygen.^35^

### Fourier-Transform Infrared Spectroscopy (FTIR) of Particles

The FTIR spectra of softwood and hardwood lignin are presented in Figures 4 and 5, respectively. A detailed description of the infrared spectra of lignin is provided in the Supporting Information.

**Figure 4:**
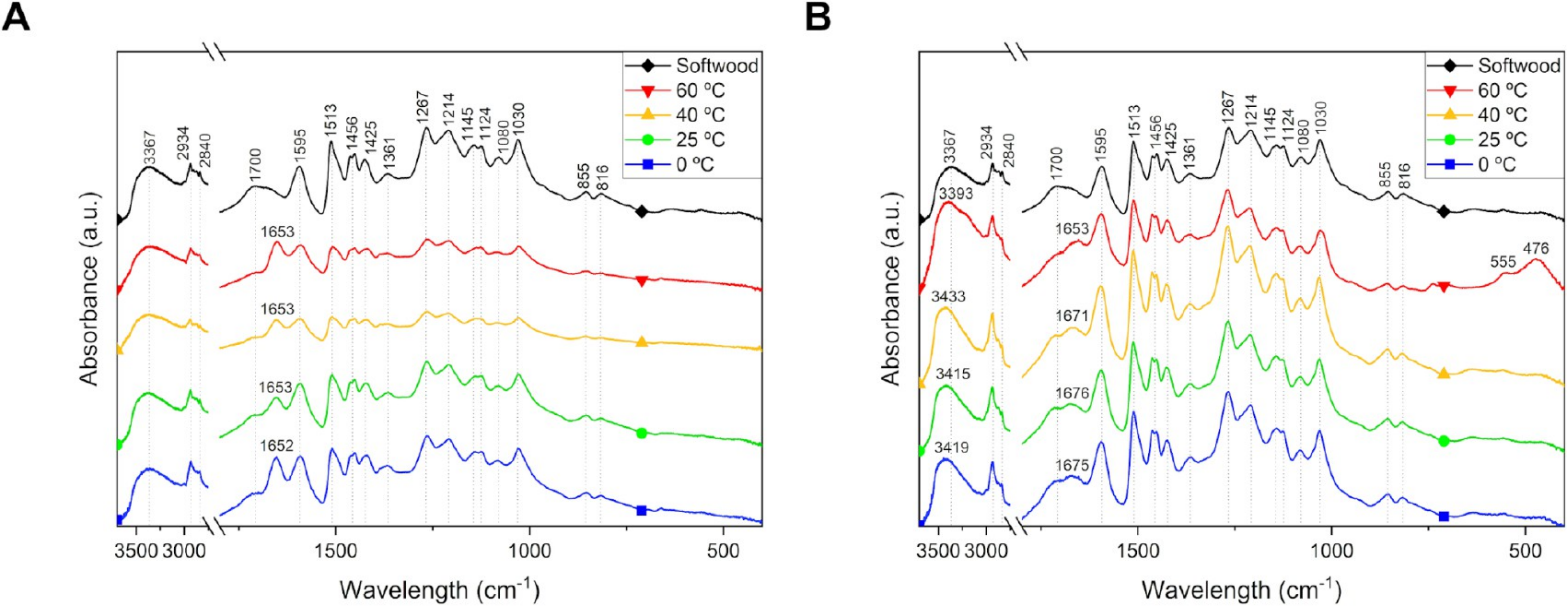
FTIR spectra of softwood lignin-originated particles : (A) DMF/water as solvent—D-SOFT0 (0 °C), D-SOFT25 (25 °C), D-SOFT40 (40 °C), and D-SOFT60 (60 °C); (B) THF/water as solvent—T-SOFT0 (0 °C), T-SOFT25 (25 °C), T-SOFT40 (40 °C), and T-SOFT60 (60 °C).

**Figure 5:**
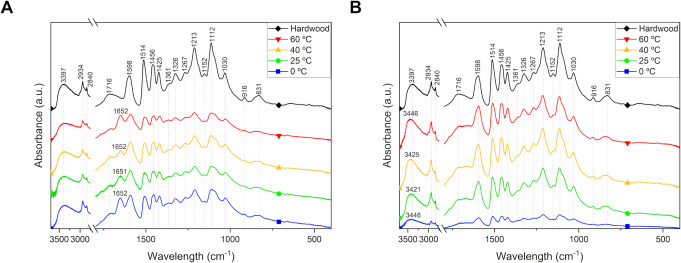
FTIR spectra of hardwood lignin-originated particles : (A) DMF/water as solvent—D-HARD0 (0 °C), D-HARD25 (25 °C), D-HARD40 (40 °C), and D-HARD60 (60 °C); (B) THF/water as solvent—T-HARD0 (0 °C), T-HARD25 (25 °C), T-HARD40 (40 °C), and T-HARD60 (60 °C).

Both softwood and hardwood lignin show the expected characteristic bands of lignin’s chemical structure. Tables S3 and S4 give a detailed correlation between each band and the corresponding chemical group. A central goal of this study is to correlate nanoparticle morphology with the conformational state of lignin molecules, as revealed by FTIR spectroscopy. The hypothesis is that the solvent quality dictates the lignin chain’s conformation, which is subsequently locked in upon precipitation.

We consider neat lignin, a solid, to be the baseline for a fully aggregated system with strong intermolecular interactions. In a bad solvent like THF, lignin is poorly solvated and collapses,^31^ mimicking its solid-state aggregation. Therefore, we predict that the FTIR spectrum of nanoparticles produced from THF should closely resemble that of neat lignin.

Conversely, in a good solvent like DMF, the polymer chains are well-solvated and adopt an expanded conformation.^31^ This disrupts the strong intermolecular interactions (e.g., π–π stacking) that are present in the aggregated state.^27^ We propose that freezing the chains in this isolated, extended state alters the collective vibrational modes of the polymer. This effect is expected to manifest as a general attenuation of absorbance bands in the 1700–1030 cm^–1^ region,^36–39^ as the cooperative interactions that enhance these peaks in the solid state are now absent.

Thus, the FTIR provides a direct probe of the polymer’s conformational state, linking the spherical particle (from THF) to a collapsed, aggregated state, and the irregular particles (from DMF) to an expanded, noninteracting state.

FTIR results in Figures 4 and 5 strongly support the proposed hypothesis. As predicted, nanoparticles produced using DMF (Figures 4a and 5a) exhibit a significant attenuation of the bands in the 1700–1030 cm^–1^ region, which is consistent with an expanded, noninteracting chain conformation. In contrast, nanoparticles produced from THF (Figures 4b and 5b) show spectra nearly identical to that of neat lignin, supporting the formation of a collapsed, aggregated state.

Additionally, a noticeable broadening of the band at 1112 cm^–1^ was observed for hardwood lignin produced in DMF. The influence of thermal energy was also apparent, as higher synthesis temperatures led to stronger spectral attenuation for softwood lignin, suggesting that temperature further disrupts the chain’s ability to form intermolecular interactions.

Beyond the effects of conformation, the spectra provide direct evidence of iron coordination. In all iron-containing nanoparticles, the high-frequency carbonyl (C=O) band was significantly altered. This band, which appears around 1700 cm^–1^ in softwood and 1716 cm^–1^ in hardwood lignin (both characteristic of ketones and carboxylic acids),^40^ was affected in one of two ways. For softwood lignin, the 1700 cm^–1^ band shifted down to the 1675–1652 cm^–1^ region. For hardwood lignin precipitated from DMF, the original 1716 cm^–1^ band was attenuated while a new band appeared at around 1652 cm^–1^. The shifting of C=O stretching frequency to lower energy is an indicator that the carbonyl groups are acting as ligands and are coordinating to the iron ions.^39,41–43^

### Scanning Electron Microscopy (SEM) of Particles

Scanning Electron Microscopy analysis was conducted to evaluate the morphology of lignin/iron nanoparticles and compare it with the morphology of lignin. Figure 6 shows that softwood lignin naturally assembles into torus-shaped particles with an internal radius of 40 ± 14 nm and an external radius of 131 ± 23 nm. Lignin with torus-shaped particles is naturally extracted from eucalyptus as a result of the assembly of low molecular weight lignin molecules into coiled structures in the presence of organic solvents.^44^ Additionally, spherical particles with a radius of 152 ± 57 nm are observed in SEM.

**Figure 6:**
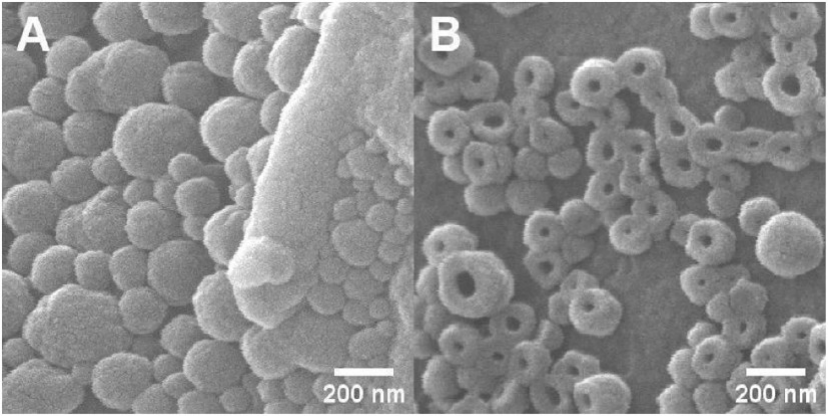
SEM images of softwood lignin: A) spherical particles and B) toroidal and sphere-with-a-hole particles. Images obtained from raw lignin dispersed in methanol and dripped on SEM support.

Both morphologies are characteristic of lignin and result from the collapse of lignin molecules upon the presence of organic solvents.^44,45^ The difference between spherical and toroidal particles is likely related to the molecular weight and flexibility of the constituent molecules. Shorter, more flexible chains rapidly collapse into compact spherical cores.

Conversely, the formation of a torus appears to be a more complex, anisotropic process. This pathway likely initiates with the self-assembly of longer, stiffer molecules into planar, disc-like aggregates. This “proto-ring” then serves as a template for the subsequent radial deposition of other molecules, a process that leads to the final torus structure.

The morphology of iron-lignin particles, as observed through SEM (Figures 7–10), depends on the solvency of the solvent, as expected and predicted by FTIR analysis.

**Figure 7:**
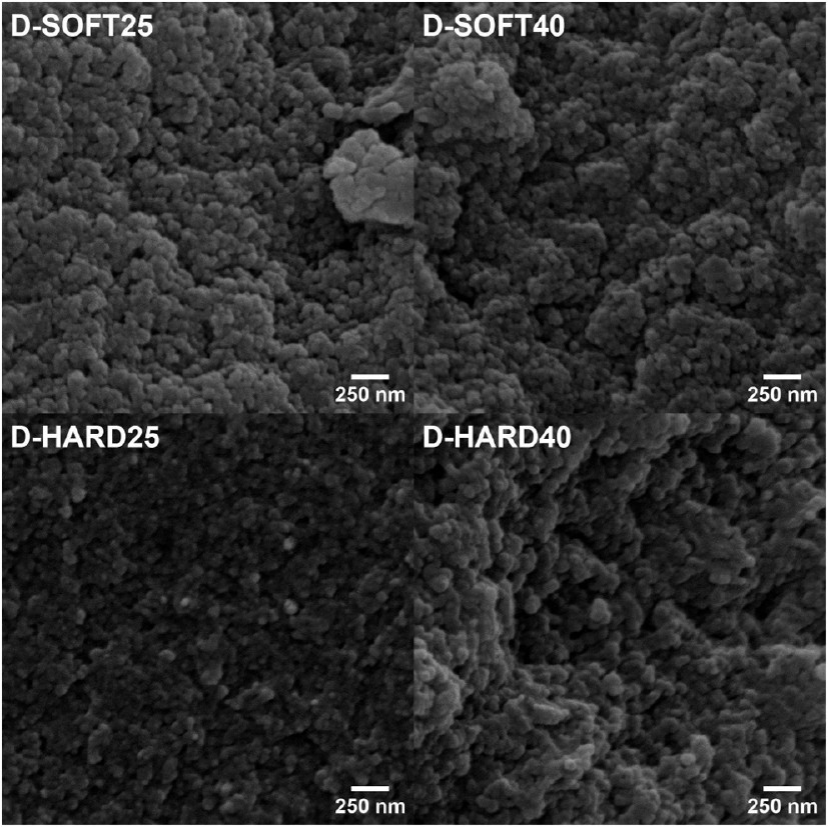
SEM images of samples originated from softwood lignin and DMF as solvent—D-SOFT25 and D-SOFT40; hardwood lignin and DMF as solvent—D-HARD25 and D-HARD40.

**Figure 8:**
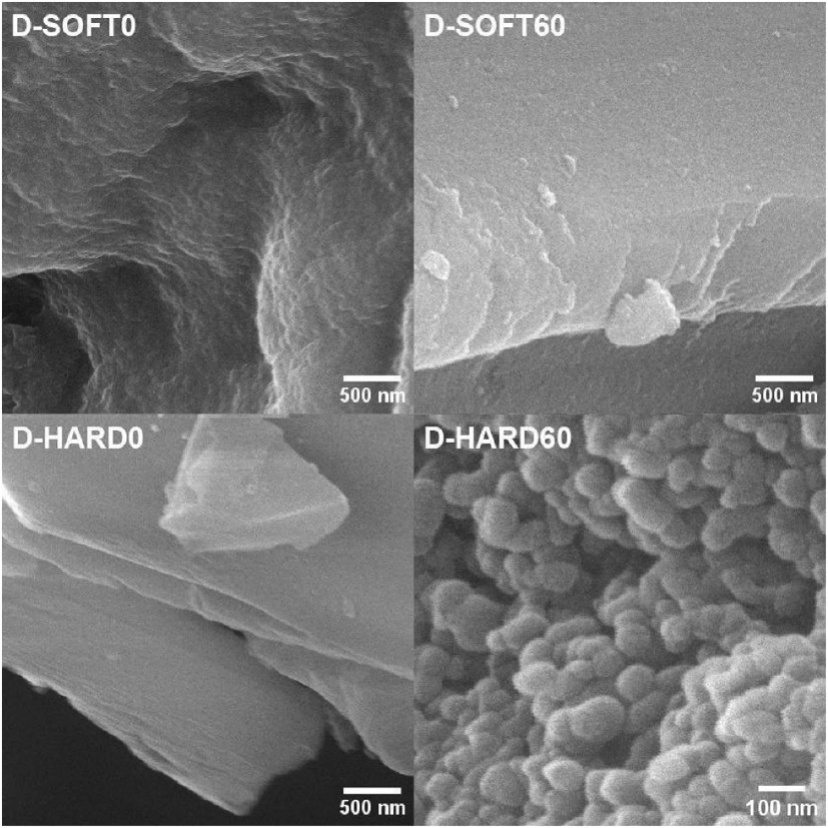
SEM images of samples originated from softwood lignin and DMF as solvent—D-SOFT0 and D-SOFT60; hardwood lignin and DMF as solvent—D-HARD0 and D-HARD60.

**9:**
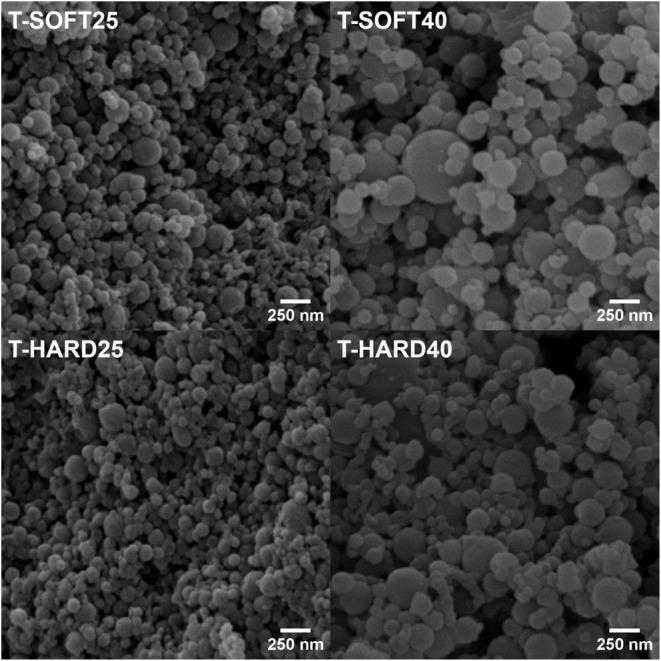
SEM images of samples originated from softwood lignin and THF as solvent—T-SOFT25 and T-SOFT40; hardwood lignin and THF as solvent—T-HARD25 and T-HARD40.

**Figure 10:**
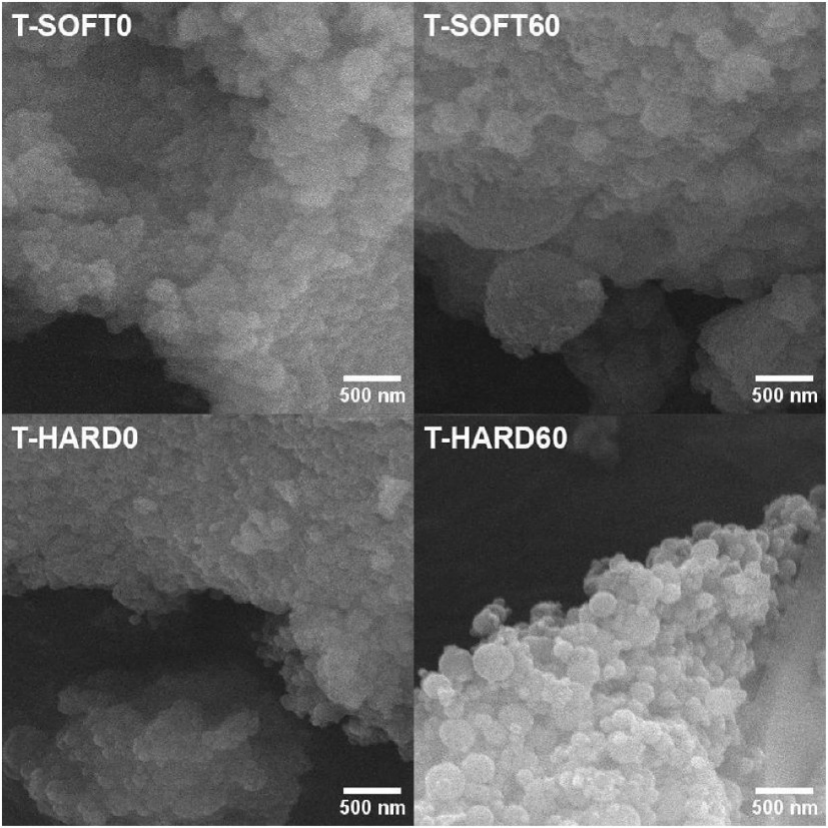
SEM images of samples originated from softwood lignin and THF as solvent—T-SOFT0 and T-SOFT60; hardwood lignin and THF as solvent—T-HARD0 and T-HARD60.

While iron-lignin nanoparticles produced using THF/water as a solvent form spherical particles at 25 and 40 °C, and spherical agglomerated particles at 0 and 60 °C, nanoparticles produced using DMF/water as a solvent (Figures 7 and 8) show shapeless agglomerated particles for both lignin types produced at 25 and 40 °C.

Moreover, at 0 and 60 °C, nanoparticles produced using DMF/water as a solvent form a bulk structure by the aggregation of shapeless structures. This bulk structure is observed for all samples produced using DMF/water as a solvent at 0 and 60 °C, except for D-HARD60 that forms spherical particles.

The diameters of samples T-SOFT25, T-SOFT40, T-HARD25, and T-HARD40 were measured by applying ImageJ on the images obtained from SEM. The results were plotted as particle size distributions (Figure 11), which were slightly skewed toward smaller particle diameters.

**Figure 11:**
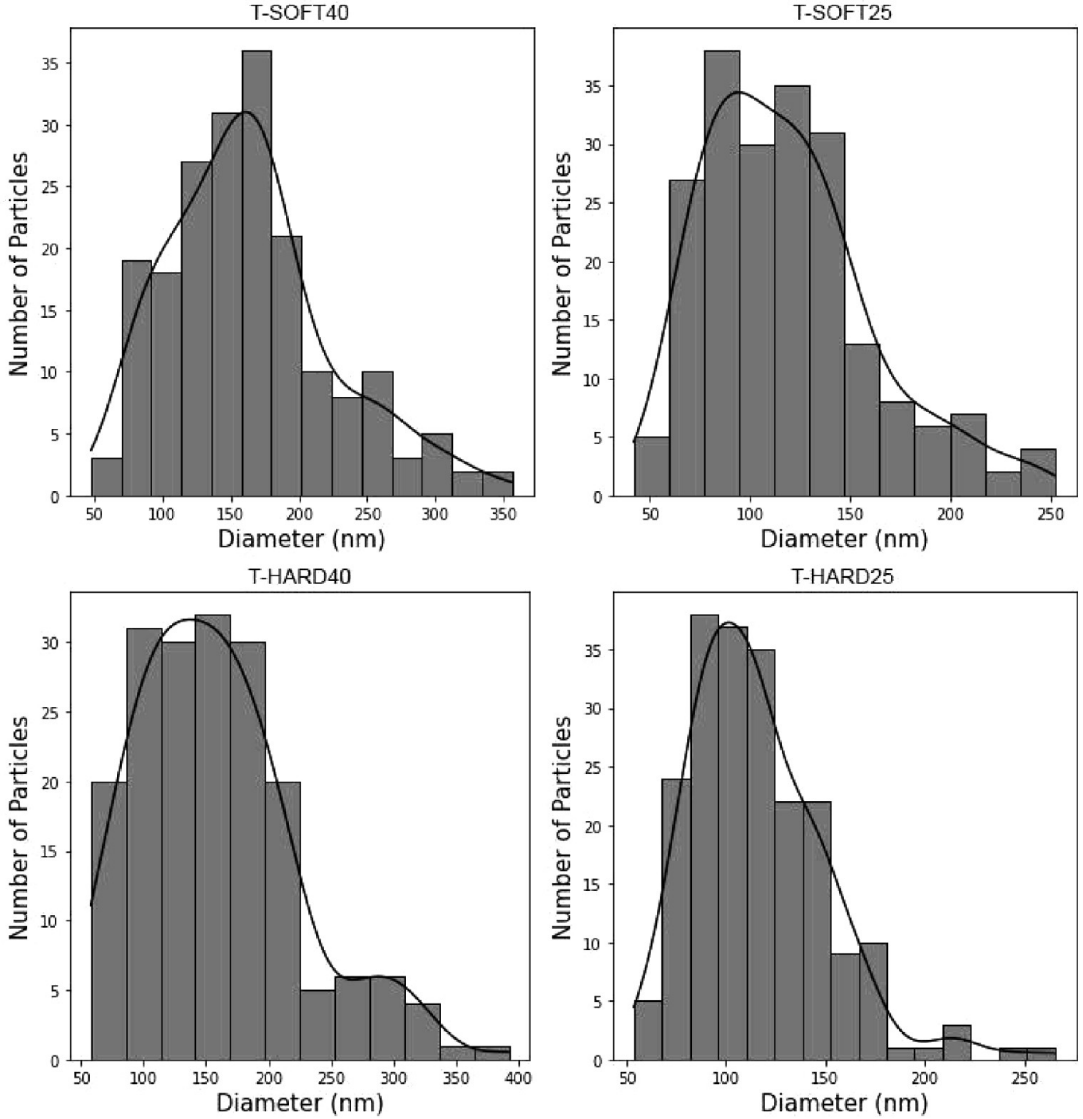
Bar chart of diameter distribution of T-SOFT40, T-SOFT25, T-HARD40, and T-HARD25 samples.

The positive-skewed tendency was confirmed by Q–Q plots shown in Figure S1. While midpoints fall on the 45-degree line, tail points tend to be dislocated above the 45-degree line. Box plots shown in Figure S2 show outliers with larger diameters in each sample and medians dislocated to the left of particle size distributions in relation to mean values.

Table 2 shows that samples T-SOFT40 and T-SOFT25 had mean diameters of 163 ± 60 nm and 118 ± 42 nm, respectively, and samples T-HARD40 and T-HARD25 showed mean diameters of 159 ± 65 nm and 115 ± 33 nm, respectively. Median values are 158 nm for T-SOFT40, 113 nm for T-SOFT25, 152 nm for T-HARD40, and 110 nm for T-HARD25. Comparing these values, an observable trend emerges: particles prepared at a higher temperature (40 °C) consistently show larger mean and median diameters than those prepared at 25 °C, while the choice of lignin type appears to have a less pronounced effect on the average particle size.

**Table 2:** Statistical Measures SEM (T-SOFT40, T-SOFT25, T-HARD40, and T-HARD25)

Sample	T-SOFT40	T-SOFT25	T-HARD40	T-HARD25
Median (nm)	158	113	152	110
Number of particles	195	206	186	209
Mean (nm)	163	118	159	115
Standard deviation (nm)	60	42	65	33
Min. diameter (nm)	47	42	58	53
Max. diameter (nm)	356	252	392	265

### Transmission Electron Microscopy (TEM) of Particles

TEM analysis was used to clarify the particle size and shape of samples that recovered inconclusive SEM results. Samples prepared at 0 and 60 °C were not conclusive regarding particle size and shape, irrespective of the solvent used. SEM images suggest that the particles produced using DMF/water as a solvent can be agglomerated but still have a spherical shape. In contrast, the particles produced using THF/water showed evident agglomeration, which made it difficult to determine particle size and shape.

Figure 12 shows images of samples produced using both DMF/water and THF/water as solvents at 0 and 60 °C. Samples D-SOFT60 and D-HARD0 were obtained using DMF as the solvent. Sample D-SOFT60 forms agglomerates of heterogeneous, shapeless particles characteristic of an extended chain structure with small black dots. D-HARD0 showed isolated particles with spaced, shapeless, stretched structures. Conversely, T-SOFT60 and T-SOFT0, produced with THF/water, resulted in spherical particles. The analysis of TEM images is in accordance with the proposed mechanism of nanoparticle formation and the influence of solvent quality on particle morphology.

**Figure 12:**
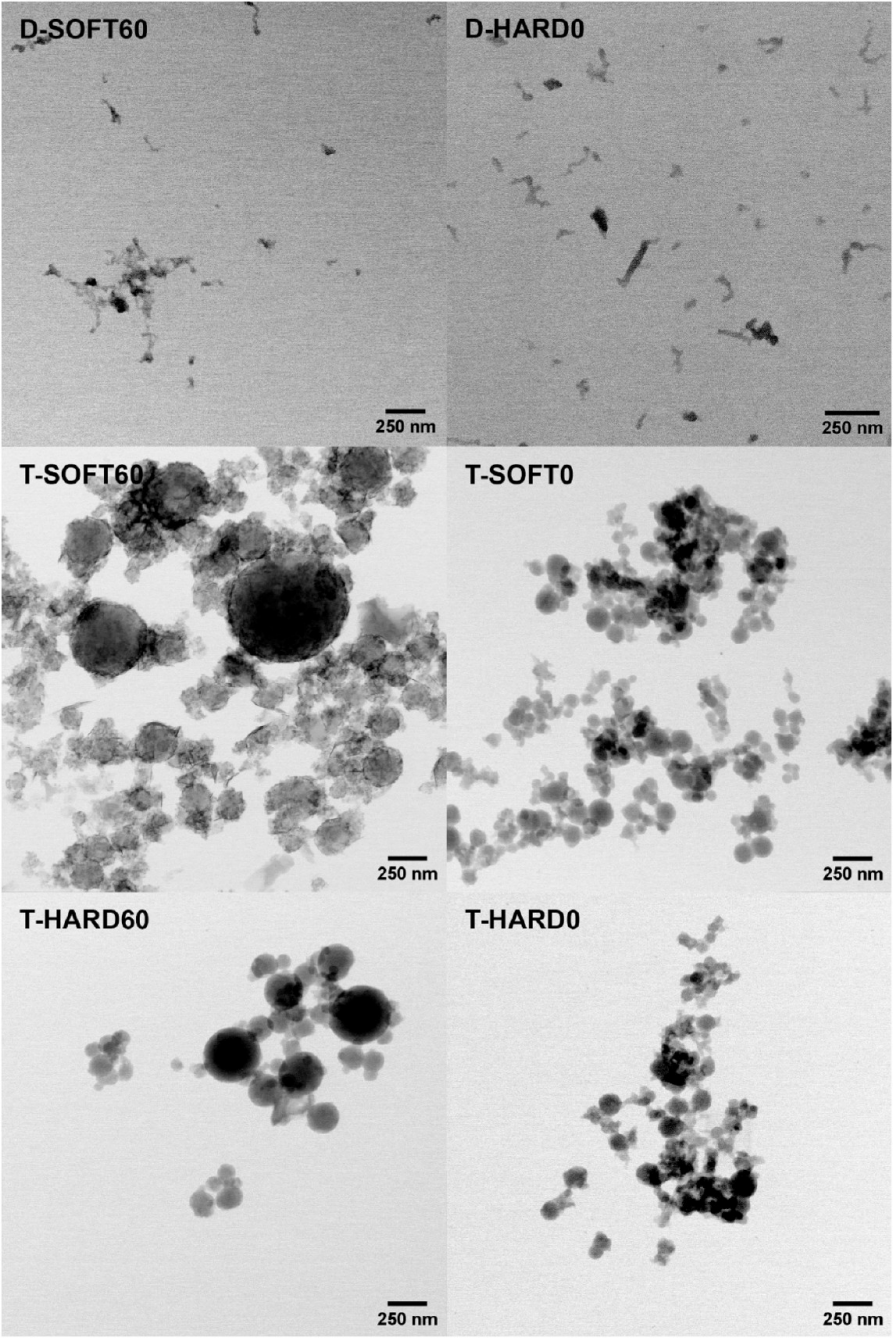
TEM images of samples originated from softwood lignin and DMF as solvent—D-SOFT60; hardwood lignin and DMF as solvent—D-HARD0; softwood lignin and THF as solvent—T-SOFT60 and T-SOFT0; hardwood lignin and THF as solvent—T-HARD60 and T-HARD0.

Additionally, the results agree with the work of Chen et al. Their work showed that DMF exhibits strong solvating capability, promoting the formation of elongated lignin structures, whereas THF, due to its weaker solvating power, favors lignin–lignin interactions, leading to the formation of spherical nanoparticles.^31^ Therefore, the use of DMF/water as a solvent in the nanoprecipitation method results in open-chain heterogeneous particles, mainly by hindering intramolecular π–π stacking and nonpolar interactions. On the other hand, intramolecular and intermolecular interactions are preserved by THF/water.^27,31^

In all samples, but especially in T-SOFT60, darker spots are observed in the TEM images. For T-SOFT60 particles, these dark spots appear on the particle surface, while in the other particles, darker regions are uniformly distributed in the particle. Recent studies on lignin and metal nanoparticles have reported the presence of small metal particles on the surface of lignin nanoparticles,^46,47^ similar to what is observed in T-SOFT60 (Figure 12). Considering the relatively high iron content detected in this sample by EDS analysis and the absence of this feature in the other samples, this dark granular appearance may indicate iron particles, possibly interacting with lignin. Moreover, sample T-SOFT0 resulted in smaller spherical particles. The same particle size variation with temperature was observed for hardwood particles, T-HARD60 and T-HARD0.

Particle diameters were measured with ImageJ using TEM images (Figure 12). Figure 13 shows a particle size distribution slightly skewed to lower particle diameters in T-SOFT60, T-SOFT0, T-HARD60, and T-HARD0 samples. The skewed tendency toward lower particle diameters was confirmed by Q–Q plots in Figure S3, confirming that result. While mid points fall on the 45-degree line, tail points tend to be dislocated above the 45-degree line. Box plots in Figure S4 show some outliers with larger diameters in each sample and medians dislocated to the left of particle distribution sizes when compared to mean values. The samples obtained at 60 °C (T-SOFT60 and T-HARD60) showed a larger average diameter when compared to the samples obtained at 0 °C (T-SOFT0 and T-HARD0). Samples T-SOFT60 and T-SOFT0 showed diameters of 126 ± 56 nm and 99 ± 34 nm, respectively. Samples T-HARD60 and T-HARD0 showed diameters of 94 ± 45 nm and 93 ± 29 nm, respectively. Samples obtained at 60 °C have larger average diameter and broader particle distribution when compared to 0 °C. T-SOFT60 had a median of 118 nm, T-SOFT0 of 96 nm, T-HARD60 of 85 nm, and T-HARD0 of 90 nm. Table 3 summarizes the results. In general, softwood lignin mean and median diameters show greater dependency on temperature than hardwood lignin particles.

**Figure 13:**
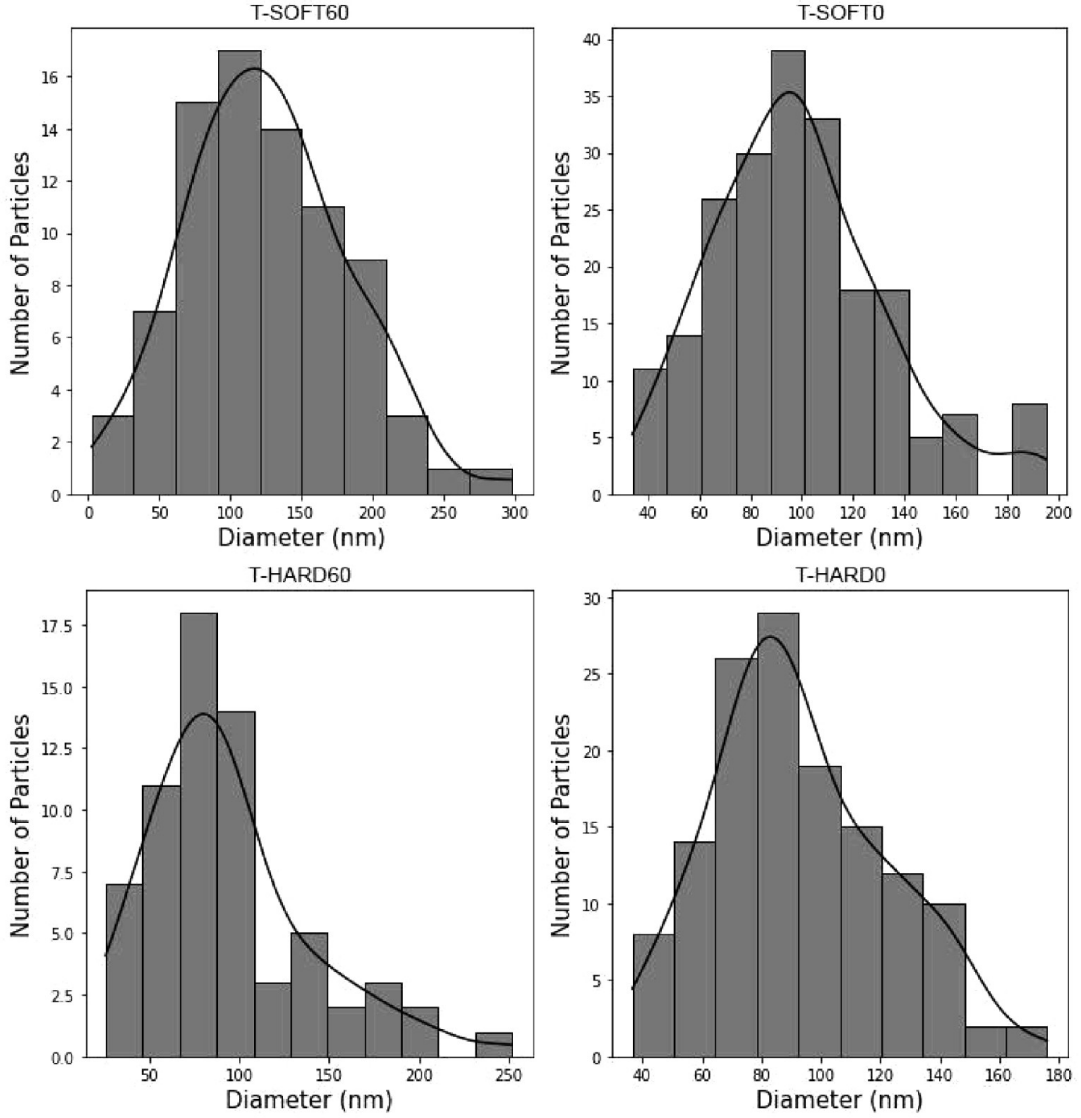
Bar chart of diameter distribution of T-SOFT60, T-SOFT0, T-HARD60, and T-HARD0 samples.

**Table 3:** Particle Distribution Size in TEM Images

Samples	T-SOFT60	T-SOFT0	T-HARD60	T-HARD0
Median (nm)	118	96	85	90
Number of particles	81	209	66	137
Mean (nm)	126	99	94	93
Standard deviation (nm)	56	34	45	29
Min. diameter (nm)	3	34	26	37
Max. diameter (nm)	298	195	252	176

Morphological analysis by SEM and TEM indicates that the use of THF as a solvent favors the formation of spherical particles, while the use of DMF results in shapeless, extended particles. Both results agree with those of Chen et al. and corroborate the mechanism that was proposed based on FTIR analysis

Additionally, the influence of temperature on particle size is evidenced by the general increase in particle diameters with the increase in temperature. Moreover, a broad particle distribution highlights the need for a particle size homogenization step to achieve greater uniformity in particle size, such as dialysis. Furthermore, it was not possible to establish a clear influence of iron content, as determined by EDS analysis (Figure 3), on the particle shape and size.

### Zeta Potential of Particles

The colloidal stability of lignin and iron-lignin nanoparticles in water was evaluated by ζ-potential analysis. The results, shown in Figure 14, do not show any direct correlation between colloidal stability and the studied variables (temperature variation or solvent quality). Figure S6 shows the original DLS curves.

**Figure 14:**
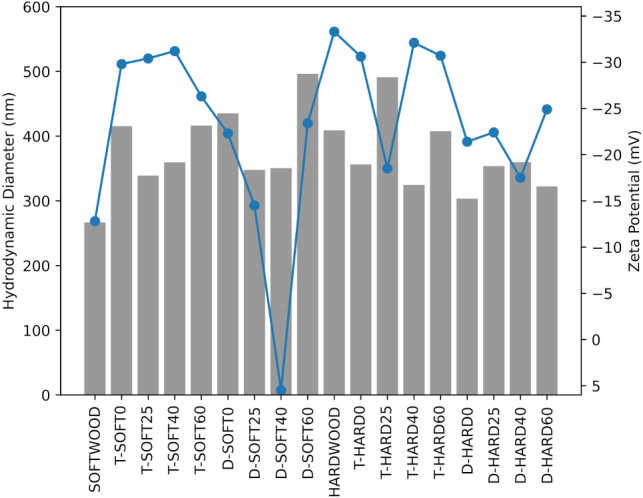
Hydrodynamic diameter and zeta potential .

Nonetheless, the absolute values of zeta potential provide insight into colloidal stability, with specific ranges indicating different behaviors.^48^ Accordingly, Table 4 demonstrates that softwood iron/lignin particles exhibited greater colloidal stability compared to neat softwood lignin – their zeta potential became more negative. In contrast, the stability of hardwood-derived particles appears to be less affected by the incorporation of iron – every hardwood sample showed a more positive value compared to the neat hardwood sample.

**Table 4:** Zeta Potential

Sample	Zeta Potential (mV)	Hydrodynamic Diameter DLS (nm)
SOFTWOOD	–12.8	266.5
HARDWOOD	–33.3	409.0
T-SOFT0	–29.8	415.3
T-SOFT25	–30.4	339.1
T-SOFT40	–31.2	359.5
T-SOFT60	–26.3	416.3
T-HARD0	–30.6	356.3
T-HARD25	–18.5	491.1
T-HARD40	–32.1	324.6
T-HARD60	–30.7	407.6
D-SOFT0	–22.3	435.2
D-SOFT25	–14.5	347.7
D-SOFT40	5.49	350.5
D-SOFT60	–23.4	496.3
D-HARD0	–21.4	303.3
D-HARD25	–22.4	353.6
D-HARD40	–17.5	359.7
D-HARD60	–24.9	322.3

Given that lignin is a negatively charged polymer due to the presence of phenolic and hydroxyl groups,^48^ all particles were expected to exhibit a negative ζ-potential. However, the D-SOFT40 sample displayed a positive value—a result that needs further investigation to be better understood.

The hydrodynamic diameter of softwood iron/lignin particles was larger than that of neat softwood, whereas the hardwood/iron particles exhibited smaller diameters compared to the neat hardwood—with the exception of T-HARD25.

It is worth noting that while neat lignin typically forms uniform particles displaying a single peak in DLS, the iron-lignin systems frequently exhibited multimodal size distributions (Figure S6). In these instances, the primary, largest peak corresponds to the mature, fully aggregated nanoparticles reported in this study. The intermittent appearance of secondary, smaller peaks is attributed to the ∼80% fraction of unreacted, low-molecular-weight lignin oligomers remaining in the suspension. Because DLS scattering intensity is heavily biased toward larger particles (I ∝ d^6^), these smaller unreacted species are only detected as distinct peaks in certain samples; in others, they likely exist as fully solvated molecular chains whose scattering signal is entirely overshadowed by the mature nanoparticles.

### Differential Scanning Calorimetry (DSC) of Particles

Ten samples of lignin nanoparticles, including neat softwood and hardwood lignin, were tested to evaluate the influence of iron ions on the lignin thermal properties.

As explained in the methodology section, the first run is needed to remove moisture, residual solvent, and thermal history. Then, the values presented in Figure 15 were obtained in two subsequent heating runs of 0 to 300 °C (Table 5) and 0 to 450 °C (Table 6).

**Figure 15:**
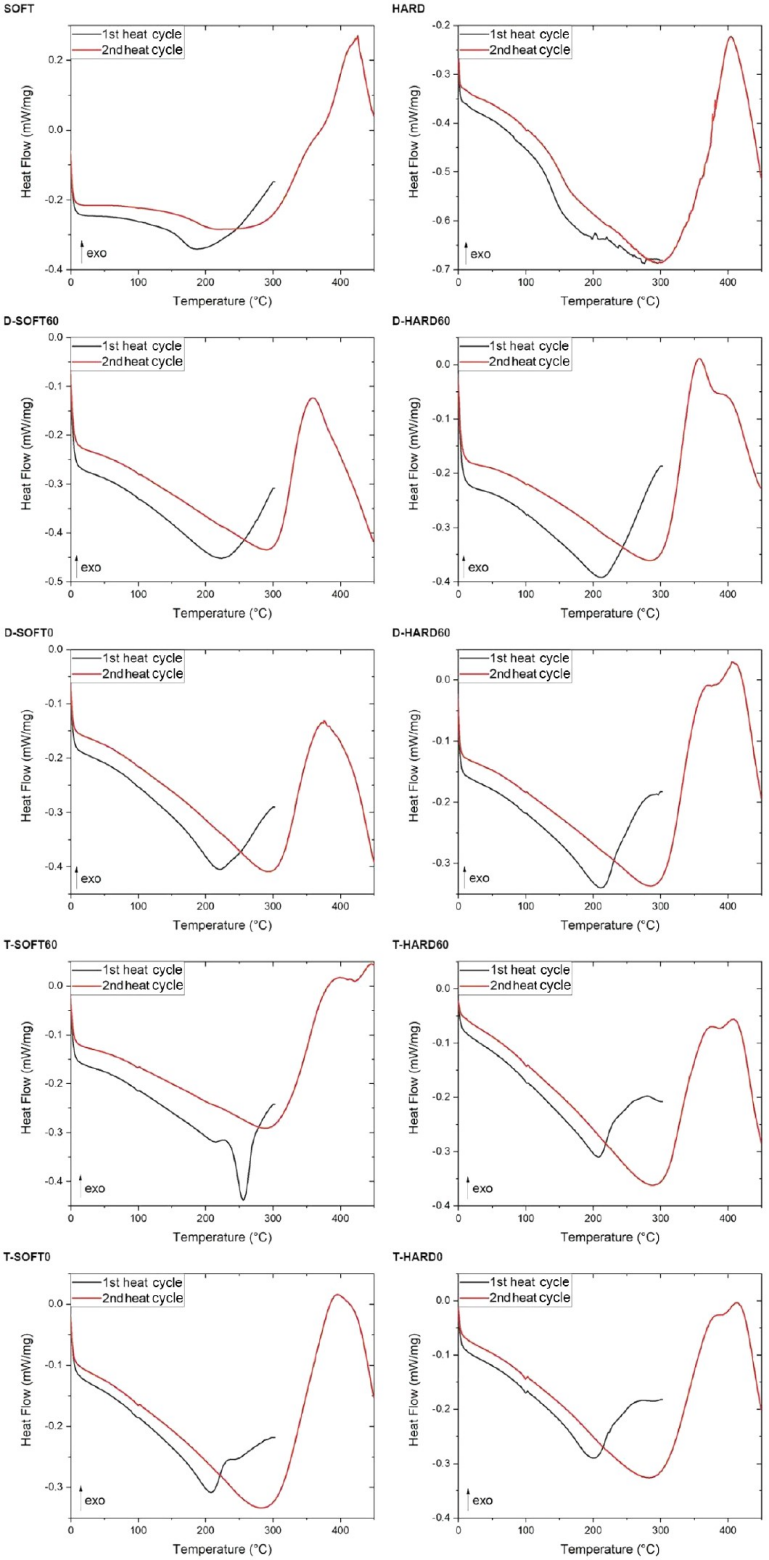
DSC analysis of precipitated particles .

**Table 5:** First Heat Run Values of DSC

Sample	Mass (mg)	*T*_g_ onset (°C)	*T*_g_ mid (°C)	*T*_g_ end (°C)
Softwood	17.7	153.6	167.8	177.8
D-SOFT60	13.5	79.3	79.6	79.8
D-SOFT0	18.3	77.9	78.2	78.5
T-SOFT60	15.11	73.5	87.3	99.7
T-SOFT0	17.3	83.9	85.7	87.5
Hardwood	7.3	122.4	138.2	155.4
D-HARD60	12.4	86.4	87.6	88.7
D-HARD0	18.9	64.9	71.4	78.0
T-HARD60	14.1	104.2	105.1	106.1
T-HARD0	13.4	102.2	102.5	102.8

**Table 6:** Second Heat Run Values of DSC

Sample	*T*_g_ onset (°C)	*T*_g_ mid (°C)	*T*_g_ end (°C)	Exothermic Peak (°C)	Pyrolysis Enthalpy (J/g)
Softwood	175.4	192.6	203.5	425.2	107.0
D-SOFT60	87.1	90.4	93.8	358.4	146.0
D-SOFT0	86.3	91.4	96.4	376.8	138.4
T-SOFT60	76.6	92.5	98.8	380.5	32.42
T-SOFT0	101.9	102.2	102.3	391.2	111.5
Hardwood	120.5	128.7	166.1	403.4	124.0
D-HARD60	78.4	82.4	86.4	356.6	150.4
D-HARD0	71.2	75.2	79.2	405.3	153.7
T-HARD60	102.6	103	103	373.7	146.1
T-HARD0	101.8	102	102	410.5	127.2

The glass-transition temperature was determined at the onset point where a change in the baseline starts. Softwood lignin exhibited a glass transition temperature (*T*_g_) of 167.8 °C, while hardwood lignin exhibited a *T*_g_ of 138.2 °C. According to the literature, the *T*_g_ values for lignin typically range from 90 to 180 °C, with the highest values generally corresponding to kraft softwood lignins.^34,49^ The variation in *T*_g_ values between the two lignin types can be attributed to differences in molecular weight, the presence of impurities, or moisture content in the samples.^50^

Overall, the *T*_g_ of lignins decreased upon incorporation of iron. Among the softwood iron-lignin samples, those prepared at 60 °C showed slightly higher *T*_g_ compared to those synthesized at 0 °C: D-SOFT60 – 79.6 °C, D-SOFT0 – 78.2 °C, T-SOFT60 – 87.3 °C, and T-SOFT0 – 85.7 °C. The decrease in *T*_g_ in hardwood iron-lignin particles, in relation to neat hardwood lignin, follows a similar tendency to that of softwood iron-lignin nanoparticles. Hardwood lignin samples synthesized at 60 °C showed higher *T*_g_ values compared to their counterparts prepared at 0 °C: D-HARD60 – 87.6 °C, D-HARD0 – 71.4 °C, T-HARD60 – 105.1 °C, and T-HARD0 – 102.5 °C.

The *T*_g_ in polymers is governed by factors such as molecular weight, chemical structure, degree of cross-linking, and intermolecular interactions.^51^ Consistent with FTIR–ATR analysis, DSC measurements suggest that the interaction of iron with the phenolic hydroxyl groups of lignin leads to a decrease in *T*_g_ due to decreased intermolecular interactions and reduced cross-linking in the lignin matrix.

During the second heating cycle, an exothermic peak begins to emerge from 300 °C onward and becomes more defined at varying temperatures, as detailed in Table 4. Broad exothermic peaks above 350 °C are indicative of lignin degradation, with the area under the DSC curve representing degradation enthalpy.^52^ Additionally, the release of sulfur dioxide between 300 and 400 °C may account for the exothermic peaks in the final DSC cycle for all samples.^53^

The DSC profiles indicate that both pure lignin and iron-modified lignin are predominantly amorphous materials. A notable exception was observed in sample T-SOFT60, which displayed an endothermic peak at 256.3 °C, suggesting a certain degree of crystallinity, potentially due to the presence of iron nitrate complexes. This peak is likely associated with the elevated iron content in the sample—exceeding 55%—as confirmed by EDS analysis of T-SOFT60. It is also important to note that the pyrolysis enthalpy values obtained in this study do not represent the complete reaction, as the analysis appears to have been terminated before full pyrolysis was achieved.

### Methylene Blue Degradation

Dye degradation is an important process for removing contaminants from water. Moreover, the study of methylene blue degradation by reactive oxygen species produced by the Fenton reaction is an important indicator of potential medical applications for lignin/iron nanoparticles.^54–56^ In this work, three iron/lignin nanoparticles were tested and compared against pure lignin in the presence of hydrogen peroxide to evaluate their ability to catalyze methylene blue degradation.

Figure S5 shows the UV–vis spectra of methylene blue for different lignin particles, and Figure 16 presents the results of relative absorbance (*C*_t_/*C*_o_) of the methylene blue peak (∼664 nm). The results show that SOFT lignin reduces MB concentration by 43%, the greatest reduction among all samples. HARD lignin is much less effective in reducing MB concentration compared to SOFT lignin. The total decrease in MB concentration by HARD lignin is 25%.

**Figure 16:**
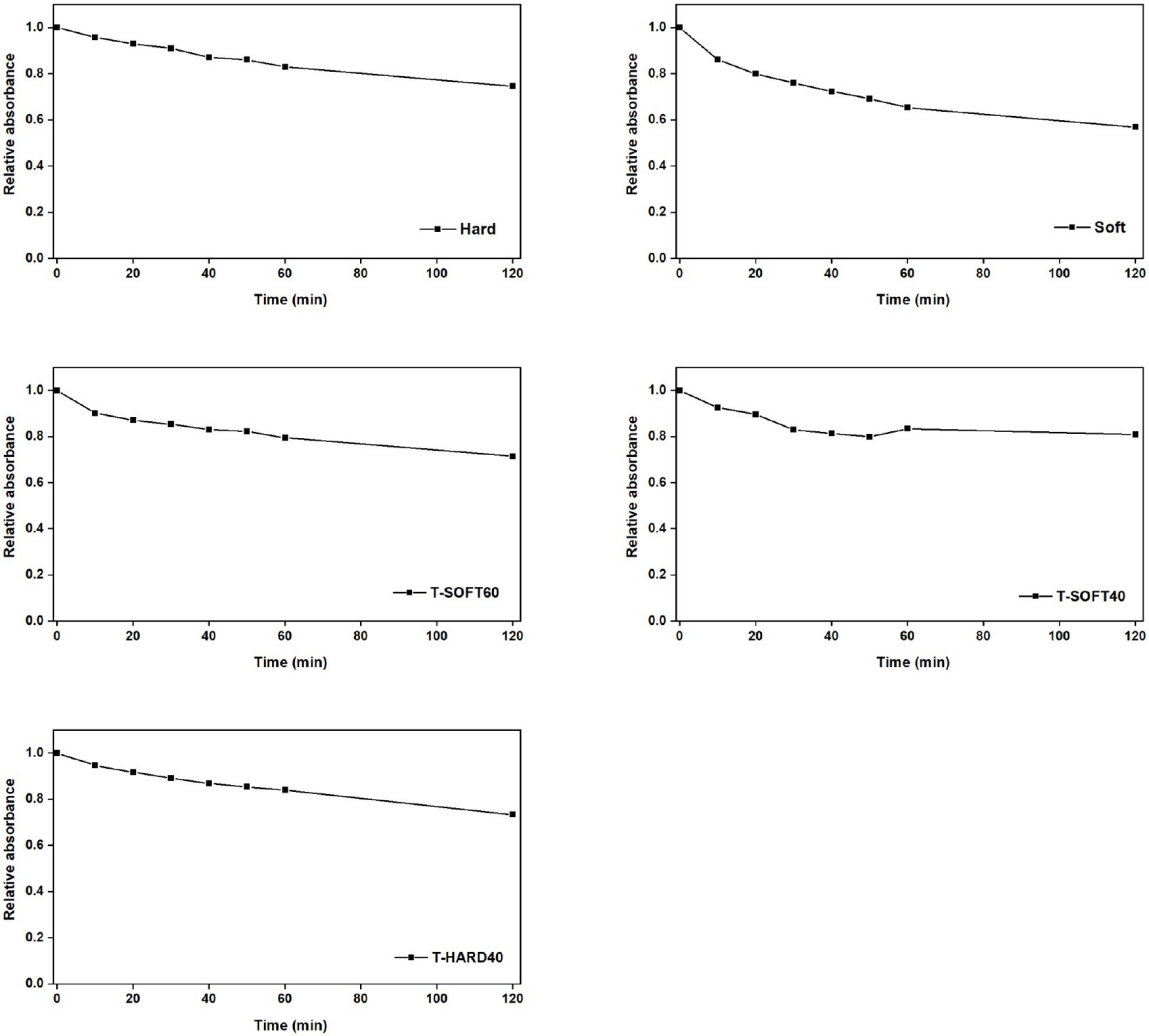
UV–vis spectra of methylene blue aqueous solutions exposed to different lignin nanoparticles.

The effect of iron/lignin nanoparticles is 2-fold. Initially, all iron/lignin nanoparticles shift the methylene blue peak from 664 to 668 nm (T-SOFT40), 669 nm (T-SOFT60), and 677 nm (T-HARD40). Moreover, over time, the peak is shifted to a higher wavelength, which could result from the formation of new compounds, which we assume results from the reaction of methylene blue and iron present in iron/lignin nanoparticles. This new peak decreases continuously over time, indicating the progression of the process.

Lignin is very effective in reducing MB concentration in solution by simple adsorption that can be explained by lignin’s high surface area and active sites. Additionally, the absence of any peak shift during neat lignin exposure to hydrogen peroxide indicates that the decrease in MB concentration is solely due to adsorption.

The photocatalytic performance of the nanoparticles is summarized in Table 7. The results show that the final position of the MB peak is different from the initial position (664 nm) and that it changes over time, except for sample T-HARD40. The final peaks are 678 nm (T-SOFT40), 686 nm (T-SOFT60), and 677 nm (T-HARD40). The observed peak shift suggests that a new substance is produced when iron-lignin nanoparticles are incorporated into the MB/hydrogen peroxide solution. Moreover, the concentration of the new substance decreased over time, possibly by adsorption.

**Table 7:** Photocatalytic Performance of Lignin Nanoparticles with Different Iron Concentrations

Sample	Variation in Methylene Blue concentration (%)	Iron Concentration(mol %)
SOFT	43	0
HARD	25	0
T-SOFT40	15	1
T-SOFT60	29	55
T-HARD40	27	0.9

Table 7 shows the variation in MB concentration calculated based on relative absorbance (ratio between absorbance at time 0 and absorbance after 120 min). It is clear from the results that lignin is more effective in reducing MB concentration than iron-lignin nanoparticles. Nevertheless, the calculation does not consider the shift observed in the main peak, which could be associated with the Fenton reaction catalyzed by iron and hydrogen peroxide.

This suggests a complex interplay between lignin and iron. While further investigation is needed, a possible explanation is that an adsorption/degradation mechanism takes place to reduce the MB concentration in solution. While neat lignin removes MB from water by adsorption, iron-lignin nanoparticles might be creating new species, leading to the peak shift, and then removing these new species by adsorption and/or degradation. Considering the key role that adsorption plays in MB removal from water, it is possible that the incorporation of iron into lignin reduces the active sites available for adsorption, leading to a smaller overall effect on relative absorbance.

Finally, the results could not establish a clear correlation between the iron concentration and MB degradation. Although the sample T-SOFT60, which has the larger iron content, is more effective than T-SOFT40, sample T-HARD40 had approximately the same efficiency as T-SOFT60 with much smaller iron content. This indicates that further research and experimentation are necessary to establish a clear relationship between the type of lignin and the iron content in the efficiency of iron-lignin nanoparticles to degrade methylene blue.

## Discussion

The results indicate that spherical lignin nanoparticles are produced from THF, while shapeless, unstructured lignin particles are produced from DMF. FTIR analysis shows that all bands from 1700 cm^–1^ to 1031 cm^–1^ change in intensity and width for the DMF particles in comparison to neat lignin, while they remain largely unchanged for THF particles. The lignin band associated with carbonyl groups (1700 cm^–1^) is consistently affected by the incorporation of iron in both systems. Additionally, temperature has a dramatic effect on the size of the lignin nanoparticles. At extreme temperatures (0 and 60 °C), the lignin nanoparticles are smaller than those produced at intermediate temperatures (25 and 40 °C). Based on these results, a dual mechanism is proposed to explain the formation of lignin-iron nanoparticles: micelle-driven nucleation for THF and solubility-driven precipitation for DMF. Figure 17 shows a schematic representation of the proposed mechanism.

**Figure 17:**
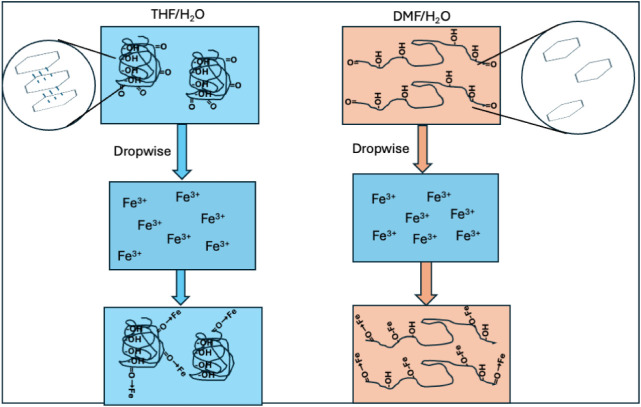
Proposed mechanism of iron-lignin nanoparticle formation by nanoprecipitation.

Initially, lignin is dissolved in both solvents, and the resulting solution is filtered to separate undissolved macroscopic fractions that are likely formed by larger, highly aggregated molecules. Because THF is a marginal solvent for lignin, the polymer molecules tend to adopt a collapsed configuration, aggregating to form micellar structures that eventually precipitate, depending on their critical aggregation concentration (CAC). Conversely, because DMF possesses a much greater solvent power for lignin, the molecules become highly solvated and are extended. In this case, the formation of lignin particles is governed strictly by the solubility limit of the extended chains.

By dropping the lignin solutions into aqueous iron solutions, the CAC and solubility limits are rapidly exceeded for the THF and DMF solutions, respectively, leading to the precipitation of lignin nanoparticles.^57^ The micellization mechanism—which requires further structural research to be definitively proven—aligns with models proposed by Vamvakaki et al.^58^ and Ievgen V. Pylypchuket al.^44^ The distinct shapes of the lignin nanoparticles can be explained by the collapsed configuration of lignin chains in THF (recovering spherical particles) versus the extended configuration in DMF (recovering unstructured particles).

These preprecipitation configurations directly influence the accessibility of lignin’s functional groups to the iron ions. For THF particles, the collapsed configuration reduces the exposure of hydroxyl groups in both the phenolic and aliphatic segments. For DMF, the extended configuration makes these groups highly accessible.^58^ Mancin and coworkers^59^ have discussed how the fluidity of the membrane formed during micelle and vesicle formation dictates the accessibility of surfactant functional groups to metal ions; kinetically “frozen” or gel-state structures limit the diffusion of metal ions to the inner core, restricting interactions primarily to external surface groups.

Our FTIR results support this kinetic restriction. For THF particles, the internal hydroxyl bonds remain intact, indicating that in the collapsed form, the apolar packing between lignin molecules is unperturbed and iron does not coordinate with these buried groups. Conversely, the consistent changes in the FTIR spectra of DMF particles between 1700 cm^–1^ and 1030 cm^–1^ suggest that the extended configuration reduces native lignin–lignin interactions, allowing iron ions to freely coordinate with the exposed hydroxyl groups. Furthermore, Knight and coworkers^60^ have shown that the introduction of metal ions can disrupt the ability of polymers to form classic vesicles or micelles. In DMF, the extensive interaction between iron and the exposed hydroxyls introduces electrostatic repulsions along the polymer backbone, which prevents the chains from folding into spherical micelles even after exposure to a strong nonsolvent (water). In THF, because the lignin is already collapsed, iron interaction is limited primarily to the external carbonyl groups, leaving the spherical micelle intact.

FTIR results also show strong evidence that iron ions bond with the carbonyl groups of lignin. Because carbonyls frequently act as end groups in lignin chains,^32^ iron effectively coordinates to the polymer extremities. The basicity of carbonyl groups makes them highly suitable ligands for iron coordination—especially compared to hydroxyl groups, which are buried in THF particles or heavily tied up in native hydrogen bonding. This rapid coordination facilitates fast precipitation, while the limited relative abundance of carbonyl end-groups explains the restricted weight concentration of iron in the final nanoparticles.

In the mechanism of iron-lignin particle formation, the massive decrease in the glass-transition temperature (*T*_g_) is driven by a synergistic combination of solvent-induced fractionation and specific metal coordination. First, the initial dissolution in the organic solvent inherently fractionates the highly polydisperse kraft lignin, isolating a specific intermediate molecular weight fraction.^39^ This effectively removes the highly cross-linked, high-molecular-weight chains that typically drive up the *T*_g_ of bulk lignin, resulting in a polymer fraction that is intrinsically more mobile and enriched in chain end-groups.^61^

Building upon this lowered baseline, the exclusive iron–carbonyl interactions serve crucial functions, particularly for the THF-derived spheres. Because our FTIR evidence indicates that iron coordinates primarily with the carbonyl end-groups at the surface of the collapsed lignin chains—while the internally shielded hydroxyl groups remain unreacted—iron does not form a rigid, internal cross-linked network within the THF particles. Instead, the coordination of bulky iron complexes strictly at the chain extremities disrupts chain-end packing and increases the free volume of the polymer network. This effectively plasticizes the structure, acting in tandem with the reduced molecular weight to drive the observed 50 °C decrease in *T*_g_. Simultaneously, these surface-bound iron ions act as interchain or intermicelle bridges, connecting the collapsed lignin molecules together to enable macroscopic precipitation without rigidly cross-linking the internal polymer backbone. Conversely, in the DMF particles, the extended chain configuration allows iron to interact more intimately with the exposed internal hydroxyl groups, fundamentally altering the intra- and intermolecular interactions and preventing this same degree of chain mobility, as evidenced by the broader changes in the FTIR spectra.

For THF, the influence of temperature on particle size is dictated by the balance between solvency power and nucleation kinetics. At 0 °C, THF’s solvency is severely reduced, which creates a high degree of supersaturation and favors an ultrafast nucleation process. Faster, more abundant nucleation results in smaller particles. At 60 °C, the solvency power of THF increases, reducing the thermodynamic drive for micelle aggregation; the resulting nanoparticles are produced from a much smaller aggregation number of lignin molecules, again leading to smaller particles. At intermediate temperatures, the optimal balance between solvent quality and chain mobility allows more molecules to aggregate per nucleus prior to precipitation, resulting in larger particles.

Ultimately, the global mechanism of these iron-lignin nanoparticles must be understood through two distinct physical chemistry frameworks: the kinetic trap and thermodynamic stability (fractionation).

The nanoprecipitation method utilized in this work involves adding a small volume of organic lignin solution into a large volume of aqueous iron solution. This rapid solvent shifting creates a highly unstable environment. In THF, this instability drives the instantaneous aggregation of the nonpolar segments to stabilize the micellar core. This dense core immediately becomes viscous and kinetically “frozen”, making it unavailable for internal iron coordination, forcing the iron to react preferentially with external carbonyl groups. This leads to the fast precipitation of spherical particles. In DMF, the rapid solvent shift creates localized supersaturation. Because the chains are initially extended, there is no time for them to undergo thermodynamic collapse into spheres. The exposed hydroxyl groups react rapidly with iron, and the resulting metal coordination electrostatically prevents micelle formation,^62^ kinetically trapping the particles as unstructured aggregates. In both cases, this incredibly fast kinetic process favors the precipitation of only the most hydrophobic (longer) lignin chains, which represent a small percentage of lignin molecules in comparison to shorter, more soluble chains that account to more than 60% of kraft lignin molecules, contributing to the low recovery,^39,63^

The low recovery of collected lignin particles indicates that a large percentage of the lignin molecules remain in solution long after the initial precipitation and throughout the subsequent 90 min of reaction. While the kinetic trap dictates morphology, the recovery is dictated by thermodynamic equilibrium. The environment created after the solvent shift thermodynamically stabilizes lower molecular weight lignin species, preventing their precipitation. Technical lignin is highly heterogeneous; longer lignin molecules possess extended nonpolar structural domains that are highly susceptible to aggregation and precipitation in the aqueous environment. Conversely, shorter chains possess a higher relative ratio of polar functional groups (hydroxyls and carbonyls) that effectively stabilize the molecules in the water/organic mixture.^44^ The addition of highly charged iron further contributes to this stabilization; iron coordination to these shorter chains creates highly solvated, electrostatically stabilized complexes in the aqueous phase. Consequently, the nanoprecipitation process inherently acts as a thermodynamic fractionation mechanism, selectively precipitating only the larger lignin molecules while stably retaining the shorter chains in the supernatant.

## Conclusions

Lignin-iron nanoparticles were successfully produced by using an adapted nanoprecipitation method. The different shapes and sizes confirmed the influence of solvent solvency, nanoprecipitation temperature, and lignin type on the morphology and properties of lignin-iron nanoparticles. Moreover, the effect of the coiling/decoiling mechanism on the shape and size of lignin nanoparticles was confirmed. Generally, the use of strong solvating solvents for lignin, such as DMF, results in shapeless particles due to chain decoiling, while the use of weak solvating solvents, such as THF, results in spherical particles likely formed by coiled lignin chains. Additionally, higher temperatures and longer lignin chains (SOFT) resulted in larger particles.

In terms of properties, the glass transition temperature of lignin is reduced by the incorporation of iron. Besides, iron-lignin nanoparticles have larger colloidal stability, as revealed by ζ-potential.

Finally, lignin-iron nanoparticles successfully reduced the concentration of methylene blue in aqueous solution in the presence of hydrogen peroxide, which indicates that those particles act like catalysts to the Fenton reaction that produces reactive oxygen species and promotes the degradation of methylene blue. The results indicate that these particles could be used in different medical applications involving reactive oxygen species.

## Supplementary Material



## Notes

Supporting Information file contains information about mass recovery (Table S1), atomic composition (Table S2), FTIR and positions (Tables S3 and S4), as well as detailed interpretation of band position and chemical structure of lignin, detailed statistical analysis (Figures S1, S2, S3, and S4), UV–vis spectra (Figure S5), and DLS original size distributions (Figure S6) (PDF)
