# Supplementary material for: Evaluation of Iron-Lignin Particles Obtained by the Nanoprecipitation Method: Influence of Process Conditions on Morphology, Structure, and Properties
Source: ACS Omega. 2026 Jun 4;11(23):33991–4009. doi: 10.1021/acsomega.6c00916 (PMC13280902; doi:10.1021/acsomega.6c00916)
Supplement: Supplementary file 1 [file ao6c00916_si_001.pdf]

SUPPORTING INFORMATIONS

EVALUATION OF IRON-LIGNIN PARTICLES OBTAINED BY NANOPRECIPTATION METHOD: INFLUENCE OF PROCESS CONDITIONS ON MORPHOLOGY, STRUCTURE AND PROPERTIES.

Jardel Machado de Lima[a], Beatriz Alves Biscola[b], Sônia F. Zawadzki[c], Luiz Pereira Ramos[a] and Daniel Eiras[a]*

[a] Federal University of Parana, Graduate Program in Chemical Engineering, Curitiba, Brazil.

[b] Federal University of Parana, Graduate Program in Materials Engineering and Science, Curitiba, Brazil.

[c] Federal University of Parana, Graduate Program in Chemistry, Curitiba, Brazil.

*eiras@ufpr.br

Sample Description

Table S1. Nanoparticles and Reagents Masses

Sample	Lignin	Temperature	Solvent	Lignin (g)	Iron Nitrate (g)	Particle (g)
D-SOFT0	softwood	0 ºC	DMF/water	0.9992	7.3313	0.3998
D-SOFT25	softwood	25 ºC	DMF/water	0.9992	7.3645	0.4813
D-SOFT40	softwood	40 ºC	DMF/water	0.9992	7.4100	0.2776
D-SOFT60	softwood	60 ºC	DMF/water	0.9992	7.3852	0.6271
D-HARD0	hardwood	0 ºC	DMF/water	1.0000	7.3384	0.7435
D-HARD25	hardwood	25 ºC	DMF/water	1.0000	7.3637	0.6014
D-HARD40	hardwood	40 ºC	DMF/water	1.0000	7.3180	0.7400
D-HARD60	hardwood	60 ºC	DMF/water	1.0000	7.3658	0.7759
T-SOFT0	softwood	0 ºC	THF/water	1.0199	7.3455	0.9305
T-SOFT25	softwood	25 ºC	THF/water	1.0199	7.3640	0.9760
T-SOFT40	softwood	40 ºC	THF/water	1.0199	7.3179	0.8673
T-SOFT60	softwood	60 ºC	THF/water	1.0199	7.3625	1.3217
T-HARD0	hardwood	0 ºC	THF/water	1.0003	7.4077	0.7012
T-HARD25	hardwood	25 ºC	THF/water	1.0003	7.3857	0.7999
T-HARD40	hardwood	40 ºC	THF/water	1.0003	7.3770	0.8017
T-HARD60	hardwood	60 ºC	THF/water	1.0003	7.4008	0.7664

Table S2. Atomic Composition Found in EDS Analysis

Sample	Carbon (%)	Oxygen (%)	Iron (%)	Sulfur (%)
D-SOFT0	66.50 ± 8.19	12.78 ± 3.94	7.73 ± 0.08	12.99 ± 0.1
D-SOFT25	70.1 ± 0.3	26.8 ± 0.3	1.5 ± 0.2	1.7 ± 0.1
D-SOFT40	70.5 ± 0.4	24.7 ± 0.4	2.7 ± 0.2	2.1 ± 0.1
D-SOFT60	74.76 ± 5.14	12.79 ± 1.92	4.87 ± 0.12	7.59 ± 0.09
D-HARD0	59.98 ± 8.22	12.85 ± 3.97	8.12 ± 0.08	19.04 ± 0.14
D-HARD25	69.2 ± 0.3	27.4 ± 0.3	1.1 ± 0.1	2.2 ± 0.1
D-HARD40	65.7 ± 0.1	30.9 ± 0.2	1.5 ± 0.1	1.8 ± 0.0
D-HARD60	59.94 ± 8.19	13.42 ± 4.17	11.85 ± 0.07	14.79 ± 0.13
T-SOFT0	71.34 ± 5.29	11.91 ± 1.98	4.10 ± 0.11	12.65 ± 0.13
T-SOFT25	71.6 ± 0.3	25 ± 0.3	1.4 ± 0.1	2 ± 0.1
T-SOFT40	71 ± 0.3	26.6 ± 0.2	1 ± 0.1	1.4 ± 0.0
T-SOFT60	28.30 ± 3.74	10.92 ± 1.97	55.02 ± 0.34	5.76 ± 0.06
T-HARD0	63.59 ± 5.54	11.48 ± 2.22	8.87 ± 0.09	16.05 ± 0.15
T-HARD25	69.4 ± 0.2	28.5 ± 0.2	0.6 ± 0.1	1.5 ± 0.0
T-HARD40	69.7 ± 0.3	27.3 ± 0.3	0.9 ± 0.1	2.1 ± 0.1

T-HARD60	69.49 ± 8.40	12.21 ± 3.94	5.74 ± 0.07	12.56 ± 0.10

Fourier-transform infrared spectroscopy (FTIR) of Particles

Table S3. Softwood Lignin Band Positions

Band Position (cm^{-1})	Chemical Group
3367	Stretching vibrations of hydroxyl groups[1, 2, 3]
2934	Stretching vibrations from methyl and methylene groups in branch chains[1]
2840	C-H stretch from methoxy ($O-CH_3$) group[2]
1596 and 1513	Vibrations of aromatic units[1]; aryl ring streching[2]
1513	Aromatic skeletal vibration[3]
1700	Carbonyl groups[1] from non-conjugated ketones, ester and carboxyl[2]
1513	Aromatic skeletal vibration[3,4]
1425	C-H deformation vibration[5] in the same plane deforming and stretching vibrations[,6]
1361	Phenolic -OH and aliphatic C-H in the methyl radical[4]
1326	Condensed syringyl (S lignin) and guaiacyl (G lignin) rings[4]
1214	C-O bond stretching vibrations in siringyl rings[3]
1124	Asymmetric stretching of C-O-C[7], C-H- bond deforming in-plane vibrations from S lignin[8]
1267	C-O stretch in guaiacol rings[3]; G lignin[8]; guaiacyl ring breathing, C-O stretching in lignin and C-O linkage in guaiacyl aromatic methoxy group[6]
1214	Sum of C-C, C-O, and C=O stretching bond vibrations[4]
1080	C–O bond deformations in secondary alcohols and aliphatic ethers in side chains[1, 2]
1145	Aromatic C–H in plane deformation[2, 4]
1030	Aromatic C-H in plane deformation[2]; deformation in the C-O bond in primary alcohols and aliphatic ethers[3]; this band can also be associated as a vibration associated with the stretching of the C-O and C-C bonds, in addition to the bending vibrations of the C-OH bond present in polysaccharides[4]
855 and 816	C-H out-of-plane vibrations[2] in positions 2, 5, and 6 of guaiacyl units[4]

Table S4. Hardwood Lignin Band Positions

Band Position (cm^{-1})	Chemical Group
3397	Stretching vibrations of hydroxyl groups[1, 2, 3]
2840	Stretching vibrations from methyl and methylene groups in branch chains[1]; C-H stretch $O-CH_3$ group[2]
1598 and 1514	Vibrations of aromatic units[1]; aryl radical vibrations from aromatic rings [2,4]
1514	Aromatic skeletal vibration[3]
1716 and 1700	Carbonyl groups[1] from non-conjugated ketones, ester and carboxyl[2]
1421	C-H deformation vibration[5] in the same plane deforming and stretching vibrations[,6]
1361	Phenolic -OH and aliphatic C-H in the methyl radical[4]
1326	Condensed syringyl (S lignin) and guaiacyl (G lignin) rings[4]
1213	C-O bond stretching vibrations in siringyl rings[3]; sum of C-C, C-O, and C=O stretching bond vibrations[4]
1112	Asymmetric stretching of C-O-C[7]; C-H- bond deforming in-plane vibrations from S lignin[8]
1326	Condensed syringyl (S lignin) and guaiacyl (G lignin) aromatic rings[4,8]
1267	Guaiacyl ring breathing, C-O stretching in lignin and C-O linkage in guaiacyl aromatic methoxy group[6]
1150	CH in-plane deformation[4]
1030	Aromatic C-H in plane deformation[2]; deformation in the C-O bond of primary alcohols and aliphatic ethers[3]; this band can also be associated as a vibration associated with the stretching of the C-O and C-C bonds, in addition to the bending vibrations of the C-OH bond present in polysaccharides[4]

The FTIR spectra of softwood and hardwood lignins are presented in Figure 4 and Figure 5, respectively. Table S3 and S4 show the position of each band, and the chemical group associated. Both softwood (3367 cm^{-1}) and hardwood (3397 cm^{-1}) lignins have bands between 3300 and 3400 cm^{-1} that can be associated with stretching vibrations of phenolic and aliphatic hydroxyl groups.[1, 2, 3] Moreover, at 2934

cm^{-1} (softwood) and 2840 cm^{-1} (hardwood) asymmetric stretching from C-H bonds in methyl and methylene groups in branch chains is observed.[1] The small band in 2840 cm^{-1} arises from the C-H bond stretching from methoxy groups.[2]

Prominent bands in 1700, 1596 and 1513 cm^{-1} (softwood) and 1716, 1598 and 1514 cm^{-1} (hardwood) arise from aryl radical vibrations from aromatic rings.[1,2,3] The bands around 1700 cm^{-1} can be also attributed to carbonyl groups from non-conjugated ketones, ethers and carboxyl.[1,2]. Furthermore, the band present at 1513 and 1514 cm^{-1} comes from aromatic skeletal vibration[3]. At 1421 cm^{-1} (hardwood) and 1425 cm^{-1} (softwood), bands are associated with C-H deformation vibration[5] in the same plane deforming and stretching vibrations.[6] The small band at 1361 cm^{-1} in both types of lignin can be attributed to phenolic -OH and aliphatic C-H in the methyl radical.[4] In both lignins, the band at 1326 cm^{-1} is characteristic of condensed syringyl (S lignin) and guaiacyl (G lignin) rings.[4]

The main difference between hardwood and softwood lignins are between 1000 and 1330 cm^{-1}. In hardwood lignin, two bands of higher absorbance are highlighted, in 1213 cm^{-1} (1214 cm^{-1} in softwood) due to C-O bond stretching vibrations in siringyl rings of S lignin[3] and sum of C-C, C-O, and C=O stretching bond vibrations.[4] The second one is in 1112 cm^{-1} (in hardwood), typical from C-H- bond deforming in-plane vibrations from S lignin[8] and asymmetric stretching of C-O-C.[7] Also, the band present at 1326 cm^{-1} in hardwood lignin indicates the presence of condensed syringyl (S lignin) and guaiacyl (G lignin) aromatic rings.[4] In softwood lignin, the biggest absorbance band is at 1267 cm^{-1} that arises from guaiacol rings, [3] present in monomeric unit guaiacyl (G lignin),[8] found in bigger quantities in softwood lignin when compared with hardwood lignin.[9] C-O stretching in lignin and C-O linkage in guaiacyl aromatic methoxy group appears around 1267 cm^{-1}.[6] Bands at 1214 cm^{-1} (softwood) and 1213 cm^{-1} (hardwood) can be associated with the sum of C-C, C-O, and C=O stretching bond vibrations.[4]

In softwood, the band at 1080 cm^{-1} is attributed to C-O bond deformations in secondary alcohols and aliphatic ethers in side chains.[1,2] Bands at 1150 cm^{-1} (hardwood) and 1145 cm^{-1} (softwood) point to CH in-plane deformation.[2,4] The band at 1030 cm^{-1} showed higher absorbance in softwood lignin and represents the deformation in the C-O bond in primary alcohols and aliphatic ethers,[3] this band can also be associated as a vibration associated with the stretching of the C-O and C-C bonds, in addition to the bending vibrations of the C-OH bond present in polysaccharides.[4] Lastly, bands in 855 and 816 cm^{-1} in softwood lignin may indicate C-H out-of-plane vibrations[2] in positions 2, 5, and 6 of guaiacyl units.[4]

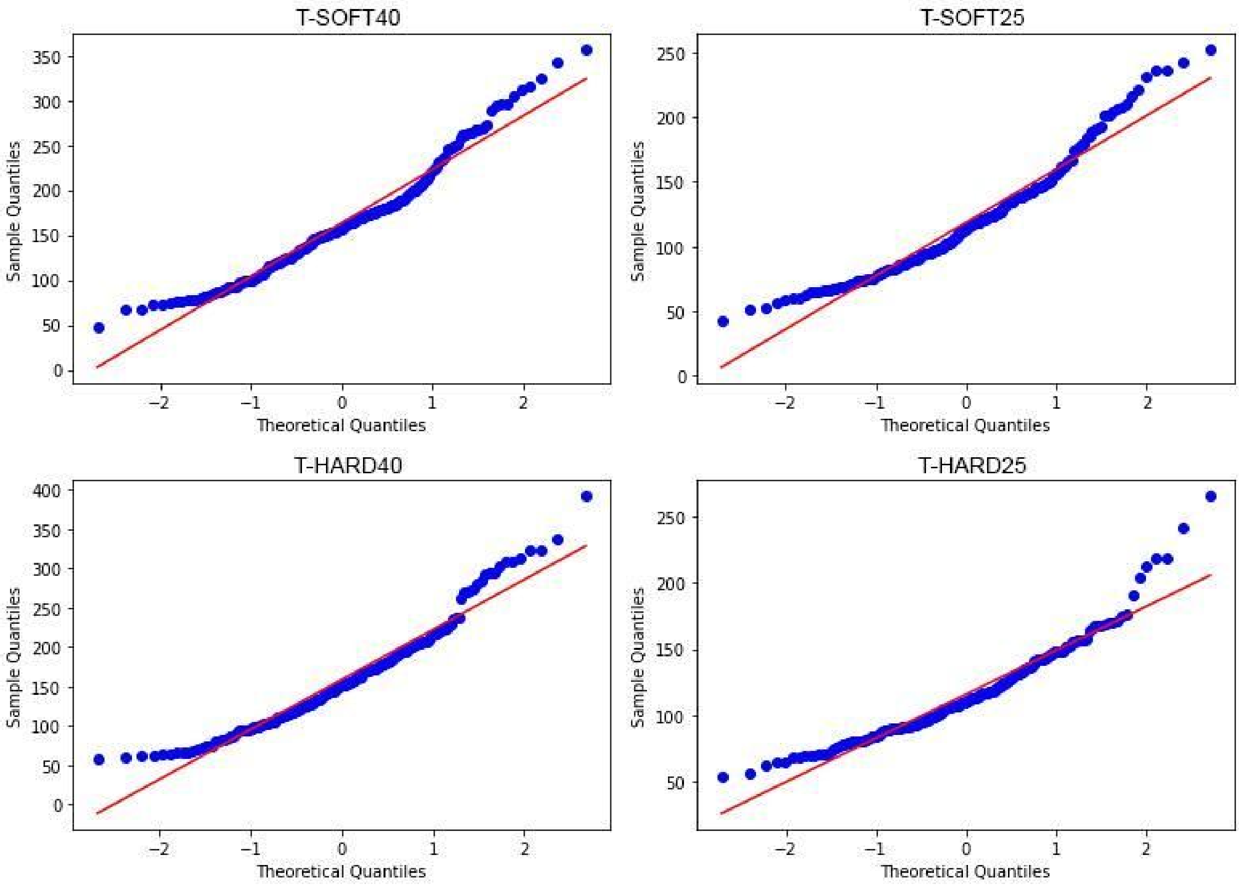

Figure S1. Normal Q-Q Plot of T-SOFT40, T-SOFT25, T-HARD40, and T-HARD25 Samples

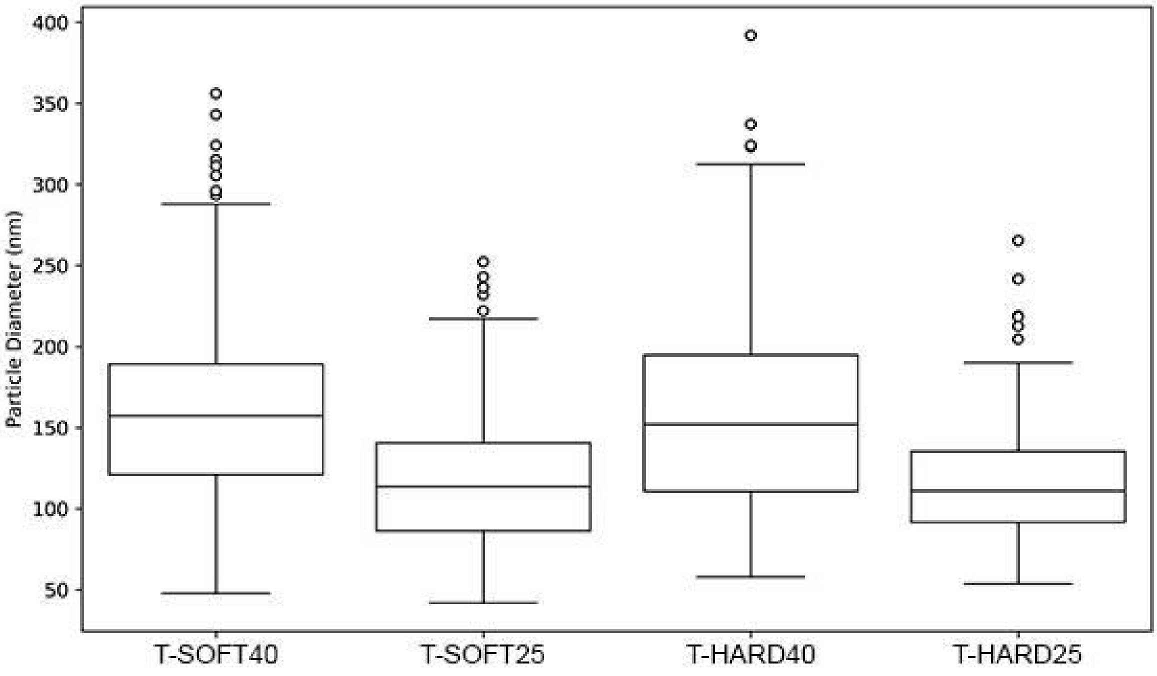

Figure S2. Box Plot of T-SOFT40, T-SOFT25, T-HARD40, and T-HARD25 Samples

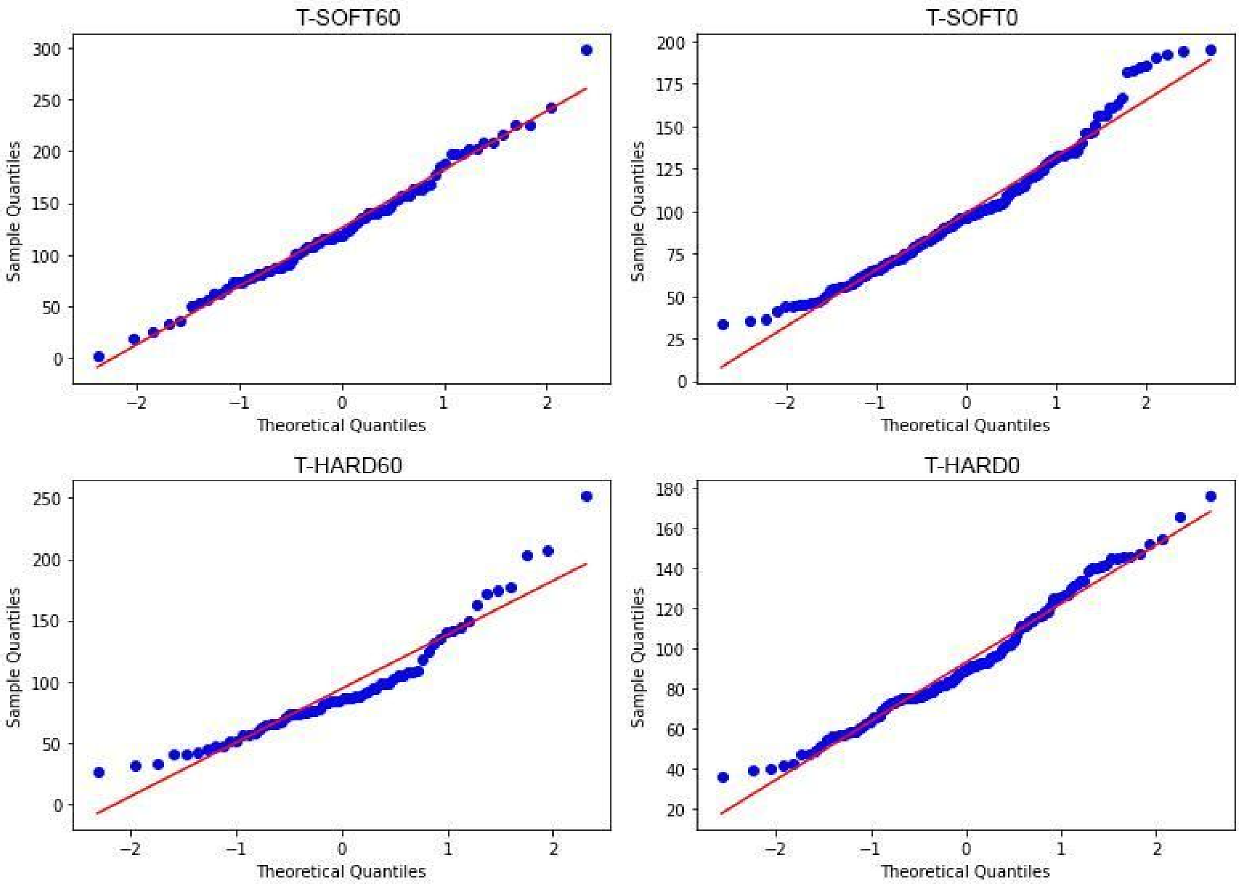

Figure S3. Normal Q-Q Plot of T-SOFT60, T-SOFT0, T-HARD60, and T-HARD0 Samples

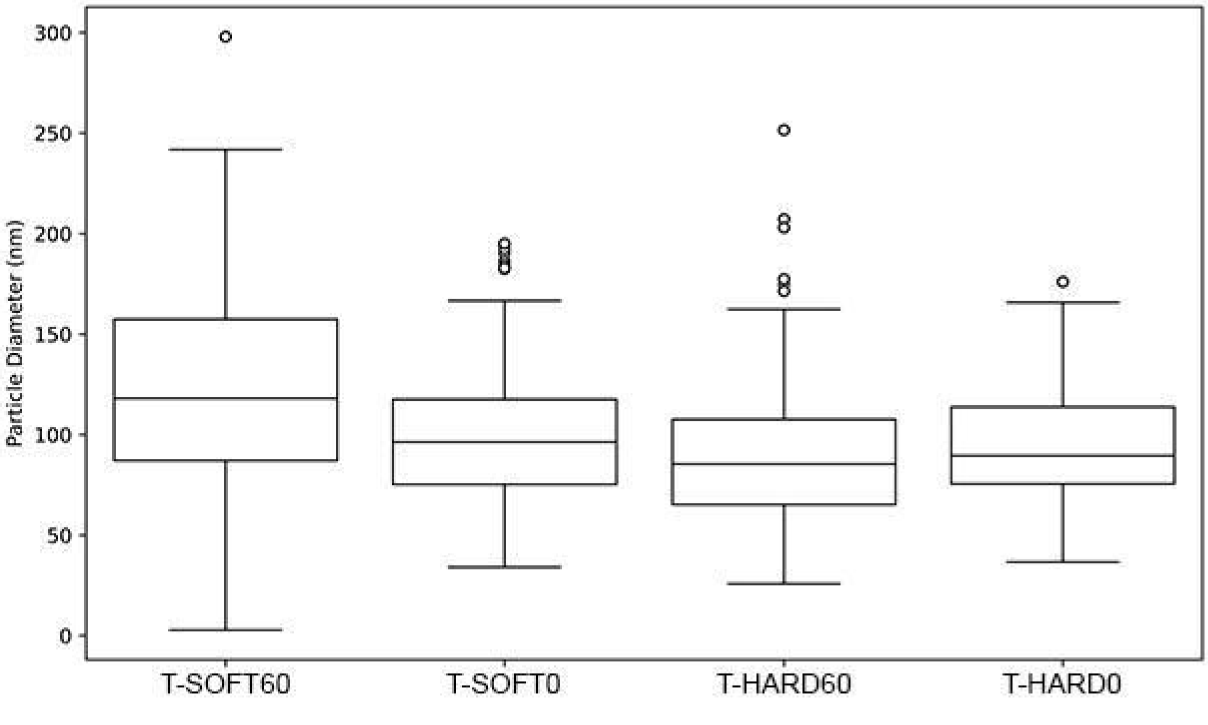

Figure S4. Box Plot of T-SOFT60, T-SOFT0, T-HARD60, and T-HARD0 Samples

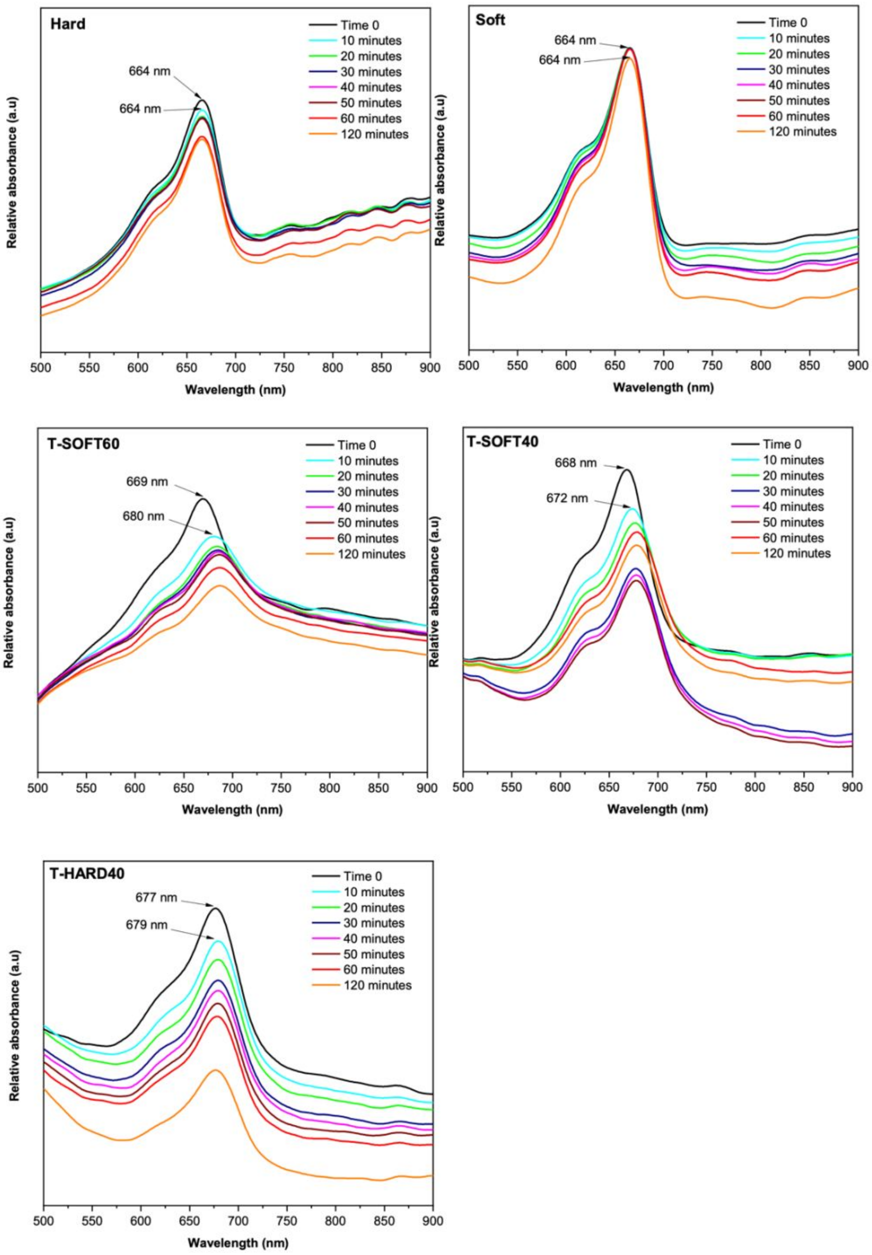

Figure S5. UV-Vis Spectra of methylene blue solutions with different lignin nanoparticles.

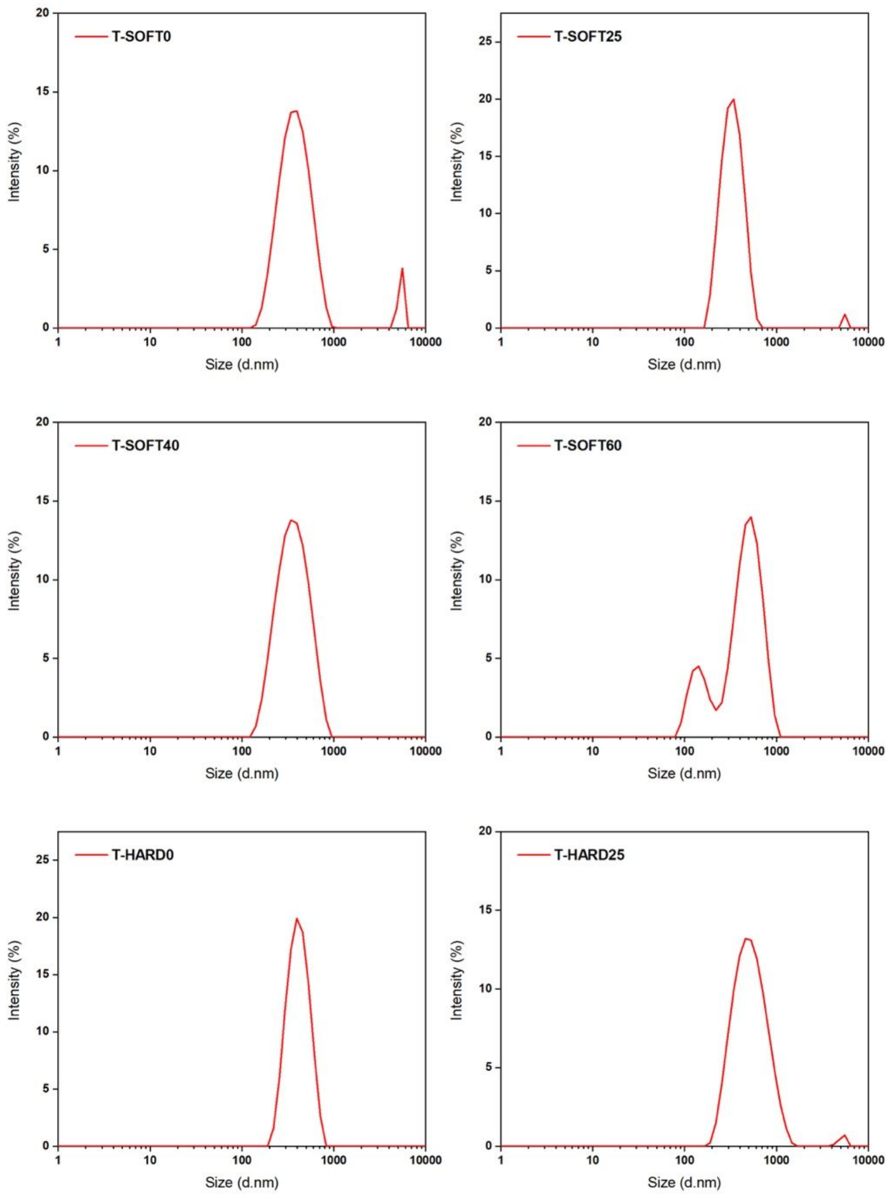

Figure S6: Original DLS curves of lignin nanoparticles in water.

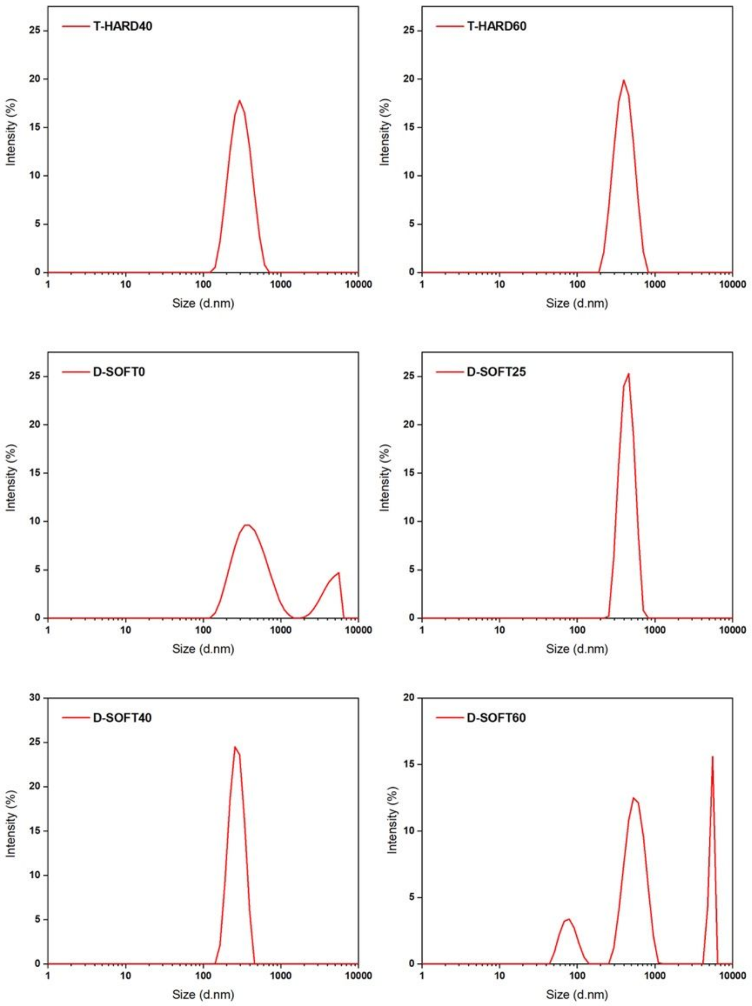

Figure S6 (cont.): Original DLS curves of lignin nanoparticles in water.

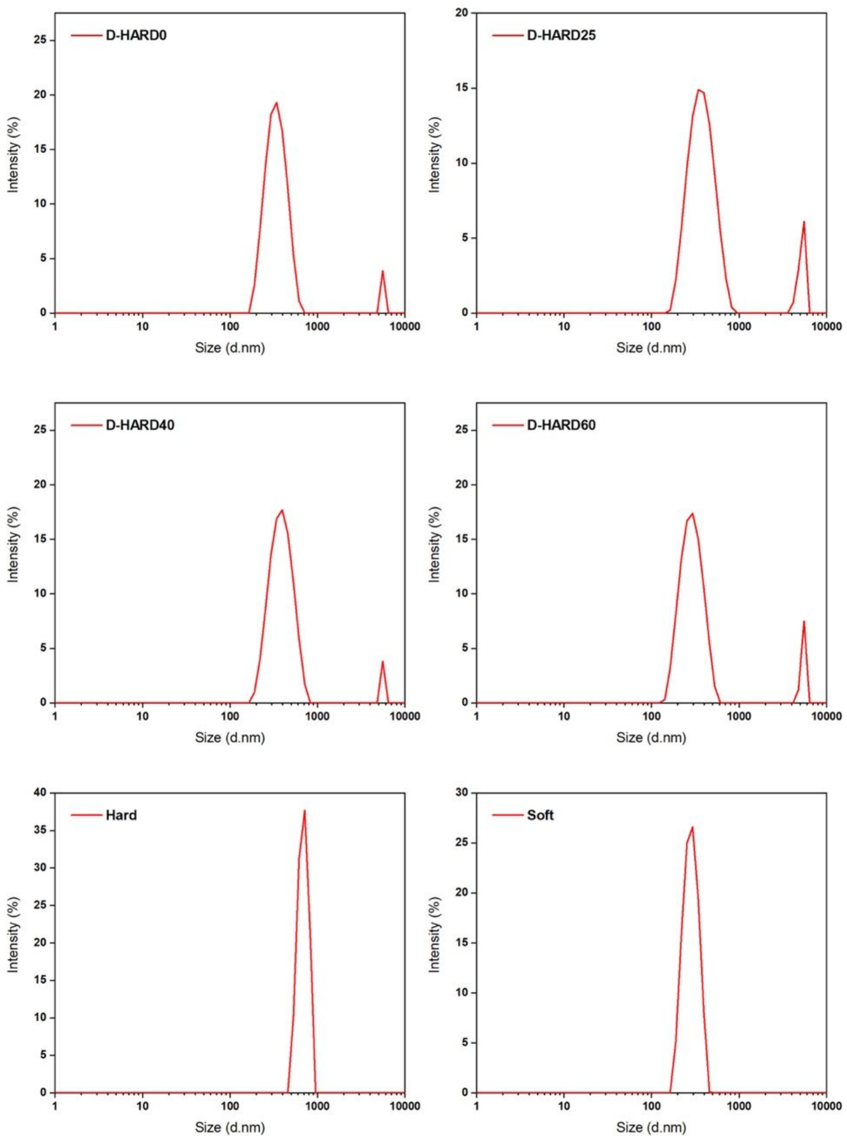

Figure S6 (cont.): Original DLS curves of lignin nanoparticles in water.

References

(1) Yan, Q.; Cai, Z. Issues in Preparation of Metal-Lignin Nanocomposites by Coprecipitation Method. Journal of Inorganic and Organometallic Polymers and Materials **2021**, vol. 31, no. 3, p. 978–996. DOI: 10.1007/s10904-020-01698-0.

(2) Stark, N. M.; Yelle, D. J.; Agarwal, U. P. Techniques for Characterizing Lignin. In: Lignin in Polymer Composites. Elsevier **2016**, p. 49–66. DOI: 10.1016/B978-0-323-35565-0.00004-7.

(3) Zhao, J.; Xiuwen, W.; Hu, J.; Liu, Q.; Shen, D.; Xiao, R. Thermal Degradation of Softwood Lignin and Hardwood Lignin by TG-FTIR and Py-GC/MS. Polymer Degradation and Stability **2014**, vol. 108, p. 133–138. DOI: 10.1016/j.polymdegradstab.2014.06.006.

(4) Boeriu, C. G.; Bravo, D.; Gosselink, R. J. A.; van Dam, J. E. G. Characterisation of Structure-Dependent Functional Properties of Lignin with Infrared Spectroscopy. Industrial Crops and Products **2004**, vol. 20, no. 2, p. 205–218. DOI: 10.1016/j.indcrop.2004.04.022.

(5) Milovanović, J.; Rajić, N.; Romero, A. A.; Li, H.; Shih, K.; Tschentscher, R.; Luque, R. Insights into the Microwave-Assisted Mild Deconstruction of Lignin Feedstocks Using NiO-Containing ZSM-5 Zeolites. ACS Sustainable Chemistry & Engineering **2016**, vol. 4, no. 8, p. 4305–4313. DOI: 10.1021/acssuschemeng.6b00825.

(6) Reyes-Rivera, J.; Terrazas, T. Lignin Analysis by HPLC and FTIR. Methods in Molecular Biology **2017**, p. 193–211. DOI: 10.1007/978-1-4939-6722-3_14.

(7) Moosavinejad, S. M.; Madhoushi, M.; Vakili, M.; Rasouli, D. Evaluation of Degradation in Chemical Compounds of Wood in Historical Buildings Using FT-IR and FT-Raman Vibrational Spectroscopy. Maderas. Ciencia y Tecnología **2019**. DOI: 10.4067/S0718-221X2019005000310.

(8) Wang, H.; Liu, Z.; Hui, L.; Ma, L.; Zheng, X.; Li, J.; Zhang, Y. Understanding the Structural Changes of Lignin in Poplar Following Steam Explosion Pretreatment. Holzforschung **2020**, vol. 74, no. 3, p. 275–285. DOI: 10.1515/hf-2019-0087.

(9) Rese, M.; van Erven, G.; Veersma, R. J.; Alfredsen, G.; Eijsink, V. G. H.; Kabel, M. A.; Tuveng, T. R. Detailed Characterization of the Conversion of Hardwood and Softwood Lignin by a Brown-Rot Basidiomycete. *Biomacromolecules* **2025**, vol. 26, p. 1063–1076. DOI: 10.1021/acs.biomac.4c01403.
